# Iron Oxidation by a Fused Cytochrome-Porin Common to Diverse Iron-Oxidizing Bacteria

**DOI:** 10.1128/mBio.01074-21

**Published:** 2021-07-27

**Authors:** Jessica L. Keffer, Sean M. McAllister, Arkadiy I. Garber, Beverly J. Hallahan, Molly C. Sutherland, Sharon Rozovsky, Clara S. Chan

**Affiliations:** a Department of Earth Sciences, University of Delaware, Newark, Delaware, USA; b School of Marine Science and Policy, University of Delaware, Newark, Delaware, USA; c Department of Biological Sciences, University of Delaware, Newark, Delaware, USA; d Department of Chemistry and Biochemistry, University of Delaware, Newark, Delaware, USA; University of California, Berkeley

**Keywords:** cytochromes, environmental microbiology, iron metabolism, iron oxidizers, outer membrane proteins

## Abstract

Iron (Fe) oxidation is one of Earth’s major biogeochemical processes, key to weathering, soil formation, water quality, and corrosion. However, our understanding of microbial contribution is limited by incomplete knowledge of microbial iron oxidation mechanisms, particularly in neutrophilic iron oxidizers. The genomes of many diverse iron oxidizers encode a homolog to an outer membrane cytochrome (Cyc2) shown to oxidize iron in two acidophiles. Phylogenetic analyses show Cyc2 sequences from neutrophiles cluster together, suggesting a common function, though this function has not been verified in these organisms. Therefore, we investigated the iron oxidase function of heterologously expressed Cyc2 from a neutrophilic iron oxidizer Mariprofundus ferrooxydans PV-1. Cyc2_PV-1_ is capable of oxidizing iron, and its redox potential is 208 ± 20 mV, consistent with the ability to accept electrons from Fe^2+^ at neutral pH. These results support the hypothesis that Cyc2 functions as an iron oxidase in neutrophilic iron-oxidizing organisms. The results of sequence analysis and modeling reveal that the entire Cyc2 family shares a unique fused cytochrome-porin structure, with a defining consensus motif in the cytochrome region. On the basis of results from structural analyses, we predict that the monoheme cytochrome Cyc2 specifically oxidizes dissolved Fe^2+^, in contrast to multiheme iron oxidases, which may oxidize solid Fe(II). With our results, there is now functional validation for diverse representatives of Cyc2 sequences. We present a comprehensive Cyc2 phylogenetic tree and offer a roadmap for identifying *cyc2/*Cyc2 homologs and interpreting their function. The occurrence of *cyc2* in many genomes beyond known iron oxidizers presents the possibility that microbial iron oxidation may be a widespread metabolism.

## INTRODUCTION

Iron (Fe) oxidation occurs in virtually all near-surface environments, producing highly reactive iron oxyhydroxides that often control the fate of carbon, phosphorus, and other metals (1). It is often assumed that abiotic reactions are the primary mechanisms of iron oxidation, particularly at near-neutral pH. However, iron-oxidizing microbes are increasingly observed in a wide range of environments, especially dark, neutral pH environments such as aquifers, soils, sediments, hydrothermal vents, and water treatment systems (2–7). Neutrophilic iron oxidizers thrive at anoxic-oxic interfaces where they can outcompete abiotic iron oxidation rates at low oxygen concentrations (8). Iron-oxidizing microbes can grow by coupling iron oxidation to the reduction of oxygen or nitrate, using this energy to fuel carbon fixation and biomass production (2, 9, 10), but in many iron-replete environments, we lack a clear understanding of the extent of microbial iron oxidation. To address this, we need to confidently identify iron oxidation mechanisms. Yet, unlike other major microbial metabolisms, we have relatively incomplete knowledge of iron oxidation pathways (11–13).

Rising interest in iron-oxidizing microbes has resulted in a surge of iron oxidizer sequencing, including isolate genomes, single cell genomes, metagenomes, and metatranscriptomes (5, 7, 9, 14–20), enabling us to search for the genes involved in microbial iron oxidation using genome mining. All sequenced genomes of the known neutrophilic chemolithoautotrophic iron-oxidizing bacteria (FeOB), the marine *Zetaproteobacteria* (*Mariprofundus* spp., *Ghiorsea* spp.) and freshwater *Gallionellaceae* (*Gallionella* spp., *Sideroxydans* spp., and *Ferriphaselus* spp.), have homologs to *cyc2* (7, 21–25), which encodes an iron-oxidizing outer membrane cytochrome first characterized in Acidithiobacillus ferrooxidans (26, 27). Many of these FeOB are obligate iron oxidizers that lack other apparent iron oxidase candidates. A second potential iron oxidase gene, *mtoA*, is found in a few *Gallionellaceae* and *Thiomonas* genomes (21, 28, 29), and functional and genetic information supports the role of MtoA and its homolog PioA in iron oxidation (30–32). However, relatively few FeOB genomes contain *mtoA*, and *pioA* is limited to phototrophic organisms, suggesting that Cyc2 is potentially a more widespread iron oxidase.

Recently, McAllister et al. presented the phylogeny of 634 unique Cyc2 homologs (7), which resolved into three distinct clusters. Two of the clusters each contain a Cyc2 homolog with verified iron-oxidizing activity—A. ferrooxidans Cyc2 (27) in Cluster 2 and *Leptospirillum* sp. strain Cyt_572_ (33) in Cluster 3. Both of these organisms are acidophilic iron oxidizers. Cluster 1 consists largely of the well-known neutrophilic iron oxidizers, including the *Zetaproteobacteria*, *Gallionellaceae*, and iron-oxidizing *Chlorobium* spp. This cluster is well supported, and these sequences are among the closest homologs to one another despite the taxonomic distance between these organisms (7). A common iron oxidation pathway for both neutrophiles and acidophiles might not be expected, due to the drastically different redox potential of Fe(II)/Fe(III) at acidic versus neutral pH (770 mV at pH 2 versus 0 ± 100 mV at pH 7 [11, 34–36]), but a conserved protein structure could suggest a shared function. To be more confident that Cyc2 is an iron oxidase in a wide range of iron oxidizers, we need biochemical verification of Cyc2 activity from neutrophilic chemolithotrophic FeOB.

Our main objective was to test for iron-oxidizing activity by Cyc2 from the well-supported Cluster 1 containing neutrophilic FeOB. We chose the Cluster 1 Cyc2 from Mariprofundus ferrooxydans PV-1, an obligate iron oxidizer, with *cyc2* as the only identified iron oxidase gene candidate in its genome (7, 37–39). Environmental metatranscriptomics of marine iron-oxidizing microbial mats dominated by *Zetaproteobacteria*, including *Mariprofundus* spp. found high expression of *cyc2*, along with evidence that *cyc2* expression may be regulated in response to Fe(II), suggesting utility for *cyc2* in an iron-oxidizing environment (7, 10). Proteomics of M. ferrooxydans PV-1 showed that Cyc2 was expressed, and a membrane complex containing Cyc2 possessed ferrocyanide oxidation activity (39). While the A. ferrooxidans Cyc2 characterization was performed in the native organism (27), neutrophilic FeOB are challenging to grow in quantities sufficient for protein assays, so we took a heterologous expression approach. We first performed structure-function predictions to inform the design of the expression constructs, which we then expressed in Escherichia coli. To test the iron oxidase function, we expressed Cyc2 from M. ferrooxydans PV-1 (Cyc2_PV-1_) under native conditions and performed iron oxidation assays and redox potential measurements of Cyc2_PV-1_ enriched by purification chromatography. We integrate structural, functional, and phylogenetic insights to explore the function of a wide range of Cyc2 homologs, including strategies for correctly identifying Cyc2 homologs and interpreting Cyc2 iron-oxidizing function.

## RESULTS AND DISCUSSION

### Cyc2 is a predicted outer membrane cytochrome fused to a porin.

**FIG 1:**
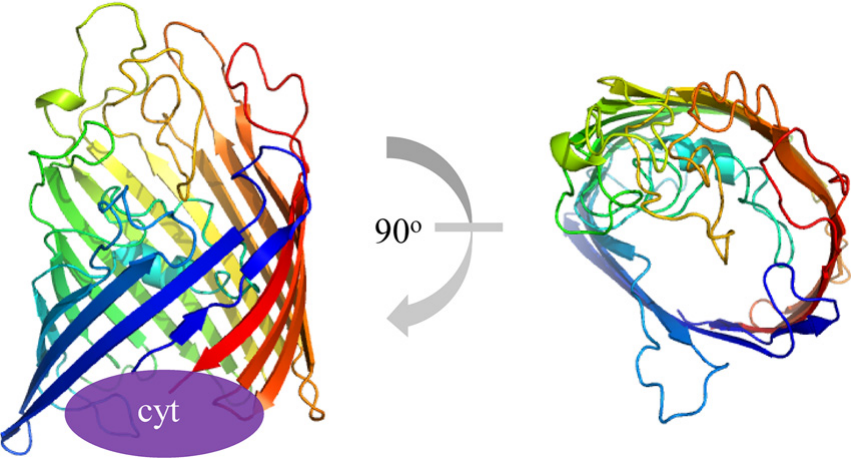
I-TASSER (49) model of Cyc2_PV-1_. Cyc2 is predicted to be a 16-stranded porin with a fused N-terminal cytochrome domain. The Cyc2 cytochrome (cyt) (purple oval) is connected to the N-terminal (blue strand) end of the porin. The view on the right is rotated 90^°^ toward the viewer to show the internal pore of the porin but does not contain the cytochrome.

We first performed a primary and secondary structure analysis to better understand the functional parts of Cyc2_PV-1_ prior to expression. Cyc2 starts with a signal peptide, predicted by SignalP (40) and has a single -CXXCH- heme-binding motif (41), placing this cytochrome in the *c*-type family. Fused to the cytochrome domain is a beta-barrel porin, based on the presence of 16 beta strands predicted by PSIPRED (42, 43) (see Fig. S1 in the supplemental material) and homology matching by HHpred (44, 45). The porin-encoding segment has low sequence homology but high structural homology to the outer membrane phosphate-selective porins OprO and OprP (PDB structures 4RJW and 2O4V [46, 47]), based on structural homology predictions by I-TASSER, MODELLER, and Phyre2 (48–51) (Fig. 1). We performed HHpred structural predictions for a diverse set of Cyc2 sequences from McAllister et al. (7), and all analyzed sequences matched to the same tertiary structure. Thus, all Cyc2 sequences are predicted to have the same structure: a 16-stranded outer membrane porin with an N-terminal cytochrome domain (Fig. 1).

10.1128/mBio.01074-21.1 FIG S1 PSIPRED prediction of secondary structure in Cyc2_PV-1_, the Cyc2 sequence from M. ferrooxydans PV-1 (D. W. A. Buchan, D. T. Jones, Nucleic Acids Res 47:W402–W407, 2019, https://doi.org/10.1093/nar/gkz297). Download FIG S1, PDF file, 0.2 MB. Copyright © 2021 Keffer et al. 2021 Keffer et al. https://creativecommons.org/licenses/by/4.0/ This content is distributed under the terms of the Creative Commons Attribution 4.0 International license.

The combination of signal peptide and beta-barrel structure suggests Cyc2 is localized to the outer membrane, as expected for a porin (52). This location is consistent with previous observations that iron oxidation occurs at the cell surface, preventing internal iron oxyhydroxide encrustation (53, 54). Porins typically possess short periplasmic turns and longer extracellular loops and have both the N and C termini in the periplasmic space (55). This standard orientation applied to Cyc2 would suggest that the cytochrome domain of Cyc2 resides on the periplasmic side of the barrel, likely as a plug at the opening of the beta-barrel pore.

### Strategy for expression of fused cytochrome-porin.

Overexpression strategies for porin proteins typically include directing expression to the cytoplasm where they form inclusion bodies that can be purified, and the porin is subsequently refolded (56). However, *c*-type cytochromes must be matured in the oxidizing environment of the periplasm to ensure covalent attachment of the heme (57). Thus, to ensure proper folding and localization to the outer membrane, our expression strategies were restricted to native conditions, at the expense of yield.

**FIG 2:**
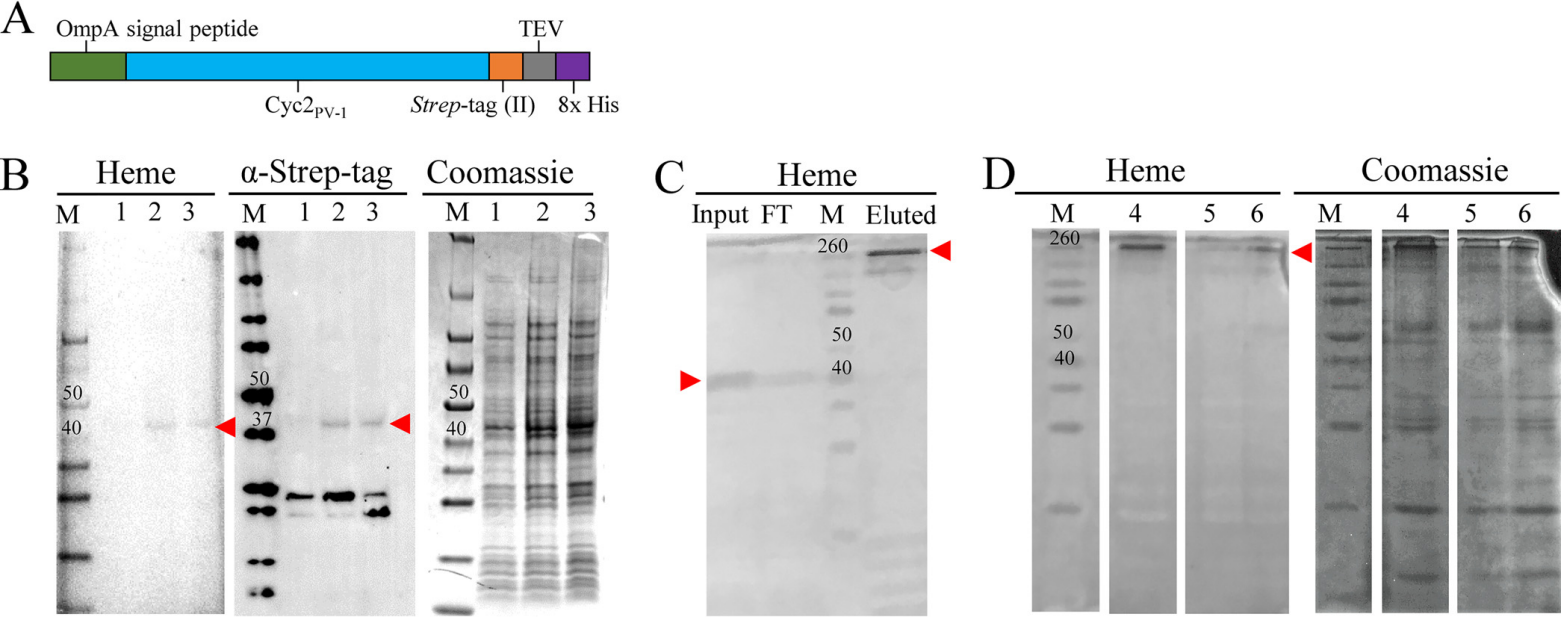
Constructs and expression of Cyc2_PV-1_. The position of Cyc2_PV-1_ is marked with a red arrowhead. The protein ladder is shown in lanes M (Spectra Broad Range on heme and Coomassie blue, WesternC on α-*Strep* tag Western blots), and relevant band sizes are labeled in kilodaltons. (A) Schematic of gene construct for expression in E. coli. (B) Representative stained SDS polyacrylamide gels showing Cyc2_PV-1_ expression in E. coli that was uninduced (lane 1), induced (lane 2), and lysed and induced (lane 3). Smaller bands visible on the *Strep* tag Western blots are nonspecific. (C) Heme-stained gel of fractions during His tag purification. After elution, Cyc2_PV-1_ migrates in a high-molecular-weight complex. (D) Stained SDS polyacrylamide gels showing Cyc2_PV-1_ enrichment fraction after His tag purification (lane 4), after cleavage of His tag (lane 5), and after concentration for assays (lane 6). Uncropped gels are shown in Fig. S2C in the supplemental material.

To maintain these functional parts during expression in E. coli C41, we synthesized a codon-optimized gene construct, and replaced the PV-1 signal peptide with the signal peptide from E. coli OmpA (56). We replaced the signal peptide to ensure E. coli directed the nascent polypeptide for translocation to the periplasm, where it could then interact with outer membrane insertion machinery (55). To assist with detection, we placed a *Strep* tag II at the C terminus where it would likely not affect cytochrome maturation (Fig. S2A). For purification, we additionally placed a linker, tobacco etch virus (TEV) protease cleavage site, and octahistidine tag (His tag) following the *Strep* tag II (Fig. 2A). In addition, we tested independent expression of the cytochrome domain and the porin domain, and while the porin was expressed, the cytochrome was not (data not shown), suggesting contacts within the full structure play a role in its stability.

10.1128/mBio.01074-21.2 FIG S2 Constructs and expression of Cyc2_PV-1_. The position of Cyc2_PV-1_ is marked with a red arrowhead. The protein ladder is shown in lane M (Spectra Broad Range on heme and Coomassie blue, WesternC on α-*Strep* tag Western blot), and relevant band sizes are labeled in kilodaltons. (A) Schematic of gene construct for expression and representative stained SDS-PAGE gels showing Cyc2_PV-1_ expression in E. coli that was uninduced (lane 1), induced (lane 2), and lysed and induced (lane 3). Smaller bands visible on *Strep* tag Western blots are nonspecific. (B) Heme-stained gel of fractions during His tag purification. Cyc2_PV-1_ migrates at its expected molecular weight in the diluted total membranes (input) and flowthrough (FT). After elution, Cyc2_PV-1_ migrates in a high-molecular-weight complex (lane 1, 300 μM imidazole; lane 2, after dialysis to remove imidazole). After TEV protease cleavage of the His tag, Cyc2_PV-1_ does not interact with the Ni-NTA column (lane 3, flowthrough; lane 4, imidazole elution). (C) Uncropped gel corresponding to Fig. 2D. See lane labels to the right of the image. Download FIG S2, PDF file, 0.2 MB. Copyright © 2021 Keffer et al. 2021 Keffer et al. https://creativecommons.org/licenses/by/4.0/ This content is distributed under the terms of the Creative Commons Attribution 4.0 International license.

### Cyc2_PV-1_ was produced by heterologous expression in E. coli.

We confirmed production of Cyc2_PV-1_ in total E. coli lysate by immunoblotting with antibodies specific to the C-terminal *Strep* tag II, as Cyc2_PV-1_ could not be identified solely by Coomassie blue staining (construct with both *Strep* and His tag [Fig. 2B]; construct with *Strep* tag only [Fig. S2A]). We confirmed that this same protein contained heme. We were unsuccessful at purification using only the *Strep* tag, but the His tag could successfully be used for enrichment of Cyc2_PV-1_ from E. coli extracts. Prior to enrichment, Cyc2_PV-1_ ran at its apparent molecular weight and was the only heme-containing protein present. After enrichment, Cyc2_PV-1_ no longer ran true to size and instead appeared as a high-molecular-weight band, possibly due to the purification conditions (high salt, high imidazole, and increased protein concentration) (Fig. 2C and D; Fig. S2B).

**FIG 3:**
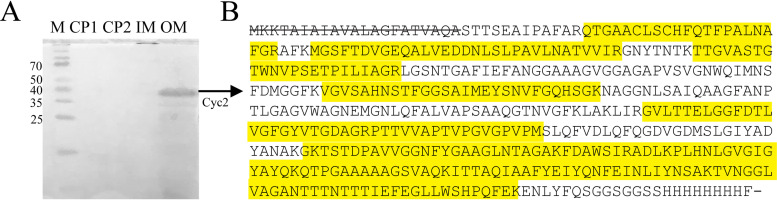
Cyc2_PV-1_ is located in the outer membrane. (A) Heme-stained SDS polyacrylamide gel of fractionated E. coli (CP1 and CP2 are cytoplasmic proteins; IM, inner membranes; OM, outer membranes; M, Spectra Broad Range protein ladder with relevant bands in kilodaltons). Cyc2_PV-1_ was found only in the outer membrane fraction (OM), and no other heme-containing proteins were seen in the OM. A Coomassie blue-stained gel is shown in Fig. S3. (B) Highlighted yellow peptides observed in tandem MS/MS confirmed the identity of the OM heme-stained band as Cyc2_PV-1_, with nearly 60% of the protein detected. The signal peptide is crossed out, as it is not present in the mature protein.

We confirmed Cyc2_PV-1_ is an outer membrane protein by using a sucrose gradient and ultracentrifugation to separate membrane components (58, 59). Cyc2_PV-1_ was found in the outer membrane fraction, in agreement with the predictions from the bioinformatic analysis (Fig. 3A). We isolated the band corresponding to Cyc2_PV-1_ from the outer membrane fraction on a heme-stained sodium dodecyl sulfate (SDS) polyacrylamide gel. Tandem mass spectrometry analysis of this band confirmed its identity as Cyc2_PV-1_, as nearly 60% of the protein sequence was detected, including regions of the cytochrome domain, the porin domain, and the *Strep* tag II (Fig. 3B; see also Table S1 in the supplemental material).

10.1128/mBio.01074-21.9 TABLE S1 Unique peptides detected by tandem MS/MS that matched to Cyc2_PV-1_ with 99% confidence. Download Table S1, PDF file, 0.1 MB. Copyright © 2021 Keffer et al. 2021 Keffer et al. https://creativecommons.org/licenses/by/4.0/ This content is distributed under the terms of the Creative Commons Attribution 4.0 International license.

**FIG 4:**
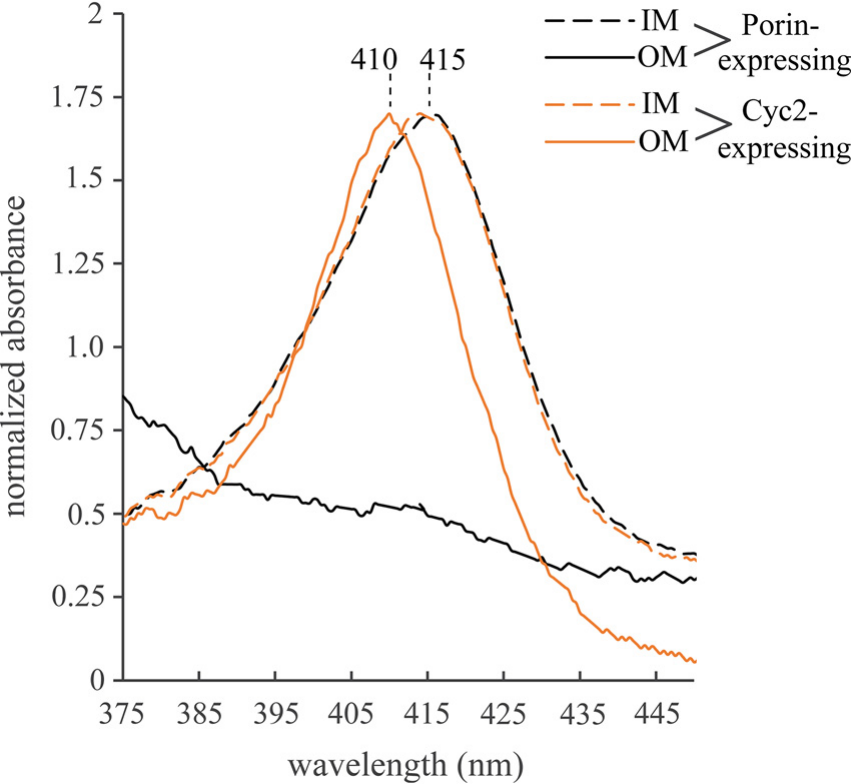
Cyc2_PV-1_ has a distinctive heme Soret peak of 410 nm. UV-Vis spectra of inner membranes (IM) and outer membranes (OM) obtained from a sucrose gradient of E. coli expressing either full-length Cyc2_PV-1_ or only the porin domain of Cyc2_PV-1_. Cyc2_PV-1_ OM had heme (orange), while porin OM did not (black), and the heme signal was distinct from other heme proteins in E. coli (IM, dashed black and orange). Heme-stained and Coomassie blue-stained SDS polyacrylamide gel and *Strep* tag Western blot are shown in Fig. S3.

10.1128/mBio.01074-21.3 FIG S3 Uncropped gels. (A) Coomassie blue-stained gel corresponding to Fig. 3A. Lanes: CP1 and CP2, cytoplasmic proteins; IM, inner membranes; OM, outer membranes; M, Spectra Broad Range protein ladder with the relevant bands in kilodaltons. (B) Gels corresponding to samples in Fig. 4. A red arrowhead indicates full-length Cyc2_PV-1_, and a blue arrowhead indicates porin only. Lanes: 1, empty vector; 2, porin lysed supernatant; 3, porin ultracentrifuged supernatant; 4, porin total membranes; 5, porin cytoplasmic proteins; 6, porin inner membranes; 7, porin outer membranes; 8, Spectra Broad Range or WesternC protein ladder; 9, Cyc2_PV-1_ lysed supernatant; 10, Cyc2_PV-1_ ultracentrifuged supernatant; 11, Cyc2_PV-1_ total membranes; 12, Cyc2_PV-1_ cytoplasmic proteins; 13, Cyc2_PV-1_ inner membranes; 14, Cyc2_PV-1_ outer membranes. Download FIG S3, PDF file, 0.1 MB. Copyright © 2021 Keffer et al. 2021 Keffer et al. https://creativecommons.org/licenses/by/4.0/ This content is distributed under the terms of the Creative Commons Attribution 4.0 International license.

### Cyc2_PV-1_ has a distinct heme spectrum.

We isolated inner and outer membranes from both E. coli expressing full-length Cyc2_PV-1_ and E. coli expressing only the porin domain of Cyc2_PV-1_. The UV-visible (UV-Vis) absorbance spectra of these two E. coli membrane fractions were compared to identify the heme signal from Cyc2_PV-1_ (Fig. 4). The inner membranes of E. coli expressing either Cyc2_PV-1_ or porin domain only were virtually identical when analyzed by UV-Vis spectroscopy, showing a heme Soret peak at 415 nm (Fig. 4, dashed black and orange spectra). These peaks represent E. coli’s native heme-containing proteins. In contrast, there are no native outer membrane heme proteins in E. coli (60). Indeed, only the outer membranes from E. coli expressing full-length Cyc2_PV-1_ had heme (Fig. 4, solid orange spectrum), with a Soret peak at 410 nm. Thus, the Soret peak at 410 nm is indicative of Cyc2_PV-1_ in our system and can be used to spectroscopically detect Cyc2_PV-1_.

### Cyc2_PV-1_ is an iron oxidase.

**FIG 5:**
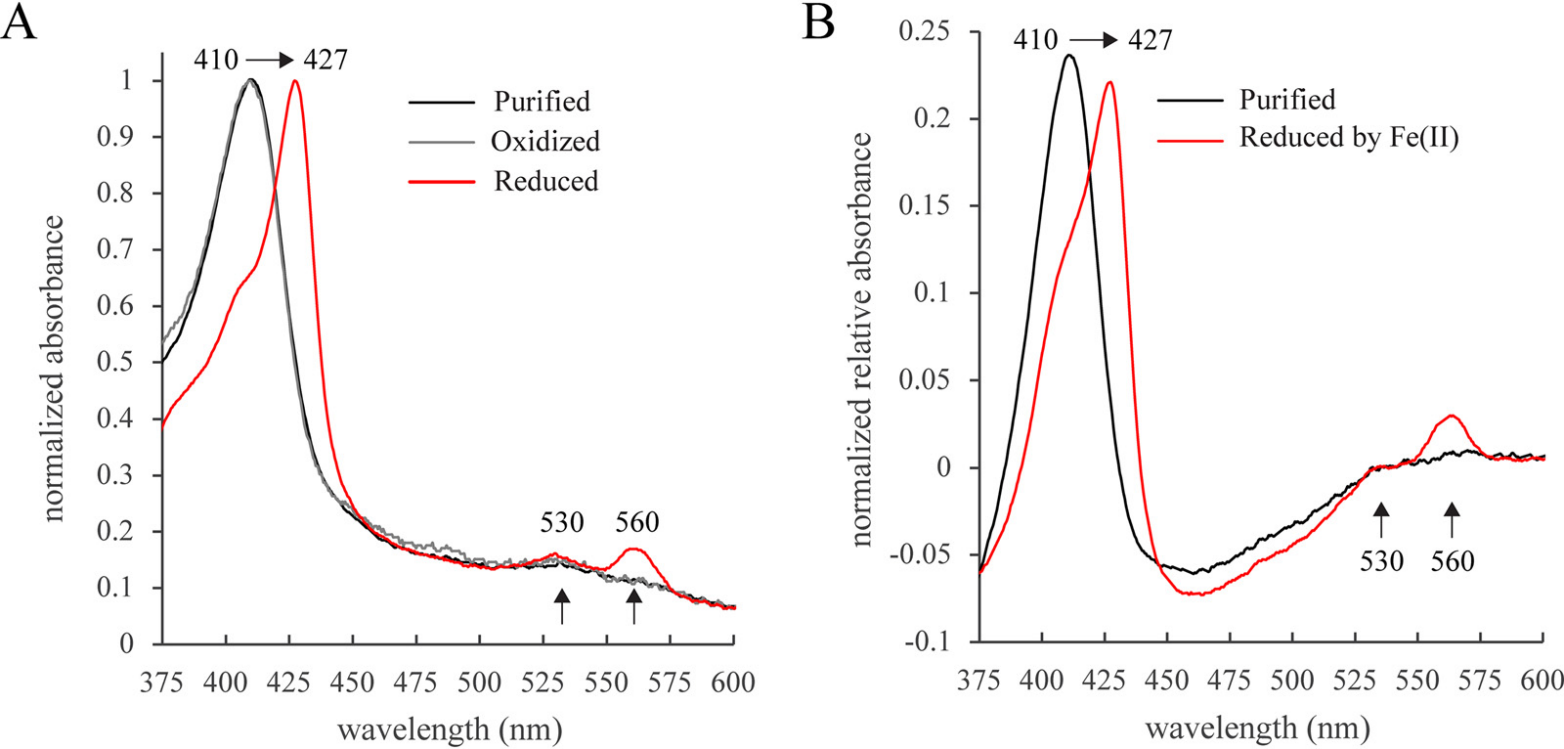
Cyc2_PV-1_ is redox active and is an iron oxidase. UV-Vis spectra of Cyc2_PV-1_ after enrichment chromatography and cleavage of the His tag. (A) Cyc2_PV-1_ as purified was oxidized (black). Addition of potassium hexacyanoferrate(III) showed no changes to the heme spectra, confirming the oxidized state (gray). Sodium dithionite reduced Cyc2_PV-1_ (red), as evidenced by the shift in the Soret peak and the appearance of the alpha and beta peaks (black arrows). (B) Cyc2_PV-1_ as purified (black). Fe(II) reduced Cyc2_PV-1_ (red), shown by the shift in the Soret peak and the appearance of the same alpha and beta peaks as the dithionite-reduced spectra.

We assayed the iron oxidation capacity of enriched Cyc2_PV-1_ by UV-Vis spectroscopy under anaerobic conditions after removal of the His tag by TEV protease cleavage. While Cyc2_PV-1_ is not pure, it is the only heme-containing protein detected, and the UV-Vis methods utilized here rely only on the heme spectra. Importantly, the heme spectrum of enriched Cyc2_PV-1_ is identical to the heme spectrum of outer membranes from E. coli expressing Cyc2_PV-1_, where Cyc2_PV-1_ was established as the only heme-containing protein (Fig. 4). Cyc2_PV-1_ could be reduced with sodium dithionite (Fig. 5A), demonstrated by the shift of the Soret peak from 410 nm to 427 nm and the appearance of α and β peaks at 560 nm and 530 nm, respectively. Addition of an oxidizing agent to enriched Cyc2_PV-1_ caused no changes, indicating that Cyc2_PV-1_ was in the oxidized state as purified (Fig. 5A). These assays demonstrated Cyc2_PV-1_ was redox active, and so we tested its capacity to oxidize Fe(II).

To perform the iron oxidation assay, we used Fe(II) with citrate to chelate the Fe(III) product, preventing formation of iron oxyhydroxide precipitates that would interfere with the UV-Vis assay. Citrate is a weak Fe(II) ligand (stability constant log *K *= 3.20; https://www.nist.gov/srd/nist46), readily releasing Fe^2+^, and has been found not to interfere with iron oxidation in FeOB (61). We calculated that ∼20% of the Fe(II) is Fe^2+^ in our assay solution (Table S2) (62), so Fe^2+^ is available as a substrate. Reaction between Fe(II) and Cyc2_PV-1_ showed the same shift of the Soret peak and appearance of the same alpha and beta peaks as reduction by sodium dithionite (Fig. 5B). These spectral changes show Cyc2_PV-1_ can accept electrons from Fe(II), demonstrating that Cyc2_PV-1_ can function as an iron oxidase.

10.1128/mBio.01074-21.10 TABLE S2 Relevant Fe(II) and citrate speciation in the iron oxidase assay buffer from Visual MINTEQ calculation (https://vminteq.lwr.kth.se/). Download Table S2, PDF file, 0.1 MB. Copyright © 2021 Keffer et al. 2021 Keffer et al. https://creativecommons.org/licenses/by/4.0/ This content is distributed under the terms of the Creative Commons Attribution 4.0 International license.

### Cyc2_PV-1_ has a redox potential of 208 ± 20 mV.

**FIG 6:**
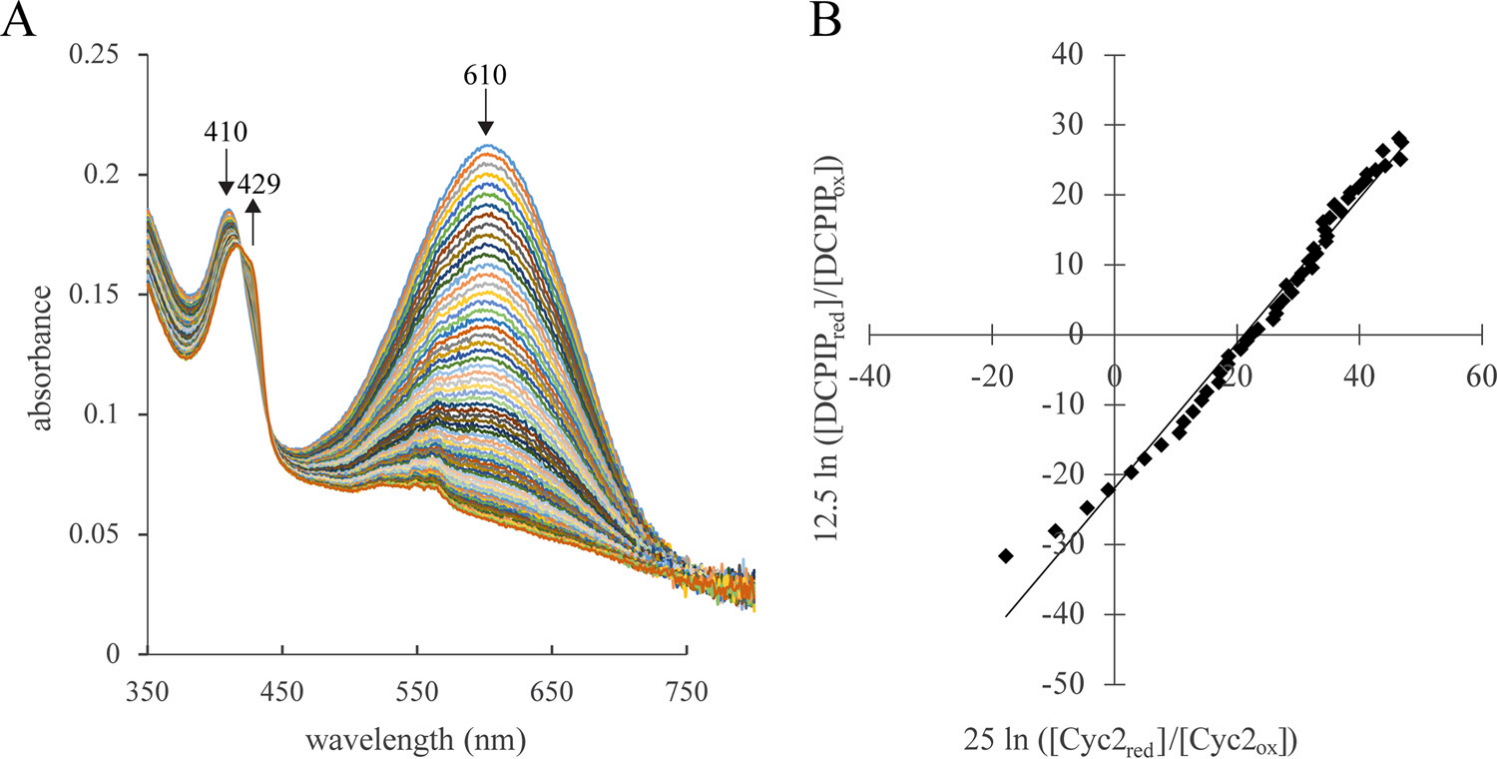
Cyc2_PV-1_ redox potential measurements shown by representative UV-Vis spectra and data plotting. (A) Spectroscopic changes observed during the determination of the reduction potential of Cyc2_PV-1_ using the dye DCPIP. UV-Vis spectra were recorded every 15 s during a xanthine oxidase-catalyzed reductive titration with xanthine, DCPIP, and Cyc2_PV-1_. (B) The ratios of the reduced and oxidized forms of Cyc2_PV-1_ and DCPIP were plotted and used to determine the redox potential of Cyc2_PV-1_.

To determine the redox potential of Cyc2_PV-1_, we used a modified Massey method protocol developed for low-yield heme proteins (63–65). Cyc2_PV-1_ was titrated against 2,6-dichlorophenolindophenol (DCPIP) with a known redox potential of +217 mV (64). By plotting the Nernst equation-transformed ratios of oxidized and reduced forms of both DCPIP and Cyc2_PV-1_ from four independent experiments (Fig. S4), we calculated the redox potential of Cyc2_PV-1_ as 208 ± 20 mV (Fig. 6).

10.1128/mBio.01074-21.4 FIG S4 Four independent redox titration reactions with Cyc2_PV-1_ and DCPIP. Download FIG S4, PDF file, 0.04 MB. Copyright © 2021 Keffer et al. 2021 Keffer et al. https://creativecommons.org/licenses/by/4.0/ This content is distributed under the terms of the Creative Commons Attribution 4.0 International license.

**FIG 7:**
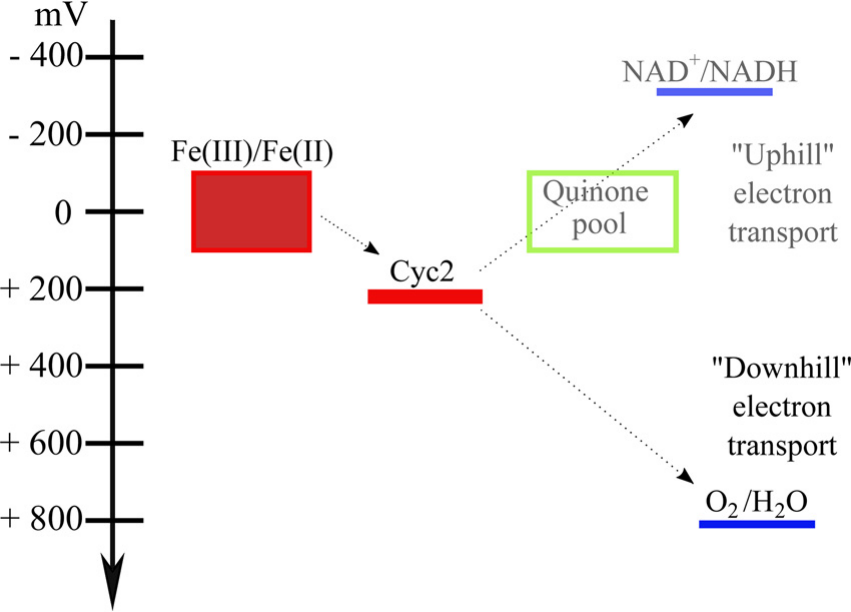
Schematic of reduction potentials showing how Cyc2_PV-1_ fits into the neutrophilic electron transport pathway. Boxes represent the range of reported values: for both the Fe(III)/Fe(II) couple (Fe^2+^/ferrihydrite couple, as predicted from FeOB mineralogy) and menaquinones and ubiquinones, this ranges from −110 to +110 mV. Much remains unknown in the “downhill” electron transport to the terminal electron acceptor, oxygen, but likely involves other cytochromes (11, 34).

Our results show a redox interaction between Cyc2_PV-1_ and Fe(II) (Fig. 5), and the calculated redox potential of Cyc2_PV-1_ (Fig. 6) puts it between the redox potentials of Fe(III)/Fe(II) (pH 7) and O_2_/H_2_O (Fig. 7), as would be expected for a neutrophilic iron oxidizer. In contrast, the redox potential for Cyc2 from *A. ferrooxidans* was measured at 560 mV (27) which is consistent with the higher Fe(III)/Fe(II) redox potential at low pH. These differences suggest Cyc2 has evolved to have appropriate redox potentials for respective FeOB environments.

### Differentiating the structure of Cyc2 from other iron oxidoreductases.

**FIG 8:**
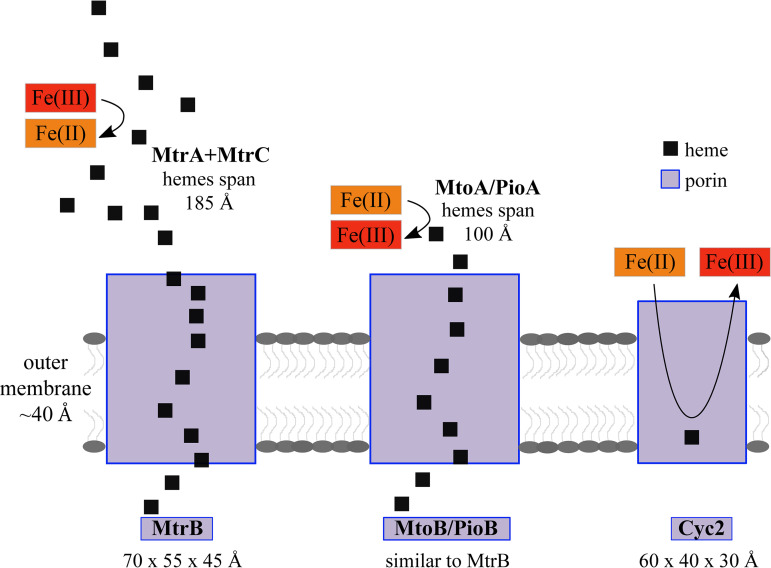
Cyc2 is different than other iron oxidoreductases. The MtrCAB complex is a 26-stranded porin with hemes spanning a range of 185 Å (67). The PioAB/MtoAB complex is likely similarly sized to MtrAB, with hemes spanning up to ∼100 Å based on modeling (32). Cyc2 is a smaller porin of 16 strands and possesses only a single heme. Due to its smaller size and placement of single heme, Fe^2+^ would have to enter the pore of Cyc2.

In total, these results bolster the evidence thus far that Cyc2 is an outer membrane iron oxidase. While Cyc2 is unique in that the cytochrome and porin are fused, its overall structure is reminiscent of other porin cytochrome complexes that play roles in iron cycling, particularly MtrCAB/OmcA and MtoAB/PioAB (30–32, 66–68). These complexes include a 26-strand beta-barrel that accommodates insertion of a decaheme cytochrome, which spans the outer membrane and may contact other extracellular decaheme proteins to conduct extracellular electron transfer (Fig. 8). In contrast, Cyc2 is predicted to have a single heme and a smaller barrel (16 strands). For organisms that eke out a living from iron oxidation, the single heme and smaller size of Cyc2 mean it requires fewer resources to produce, making it a streamlined alternative to larger porin cytochrome complexes (69).

If the pore size of Cyc2 is similar to the structurally homologous phosphate porins, the internal diameter is expected to be ∼3.5 Å (46). A model of the Cyc2_PV-1_ cytochrome domain is ∼20 × 20 × 10 Å (Fig. S5), which would fit as a plug within the periplasmic opening of the barrel, but not span the outer membrane through the porin, nor fully reside within the middle of the pore. Given our structural predictions, the cytochrome would be on the periplasmic side of Cyc2, which would allow transfer of electrons to a periplasmic component of the electron transport chain. While long-range electron transport is possible, the rate of electron transport decreases exponentially with distance (70). The hemes within MtrA are 3.9 to 6.5 Å apart (67), and similar configurations are found in other cytochromes as well, suggesting this distance is optimal for efficient electron transport. In our model, Fe^2+^ would need to enter the Cyc2 pore to some extent, possibly with the help of a chaperone or ligand that could also escort Fe^3+^ from the pore before formation of Fe(III) oxyhydroxides that would detrimentally encrust the protein. The requirement for iron to enter the pore would suggest that Cyc2 is an oxidase of dissolved Fe^2+^, distinguishing it from a multiheme iron oxidase like MtoA that could directly conduct electrons from a solid surface.

10.1128/mBio.01074-21.5 FIG S5 Three views of the modeled cytochrome domain of Cyc2_PV-1_. The view on the right is rotated 90^°^ away from the viewer compared to the view in the center. Hydrophobic residues are colored gray, and polar residues are colored red. Heme (not pictured) is covalently attached to cysteine residues (yellow) and coordinated by histidine (blue). The model was generated using MODELLER (B. Webb, A. Sali, Curr Protoc Bioinformatics 54:5.6.1−5.6.37, 2016, https://doi.org/10.1002/cpbi.3). Download FIG S5, PDF file, 0.1 MB. Copyright © 2021 Keffer et al. 2021 Keffer et al. https://creativecommons.org/licenses/by/4.0/ This content is distributed under the terms of the Creative Commons Attribution 4.0 International license.

### Recognizing *cyc2*/Cyc2 in other organisms.

**FIG 9:**
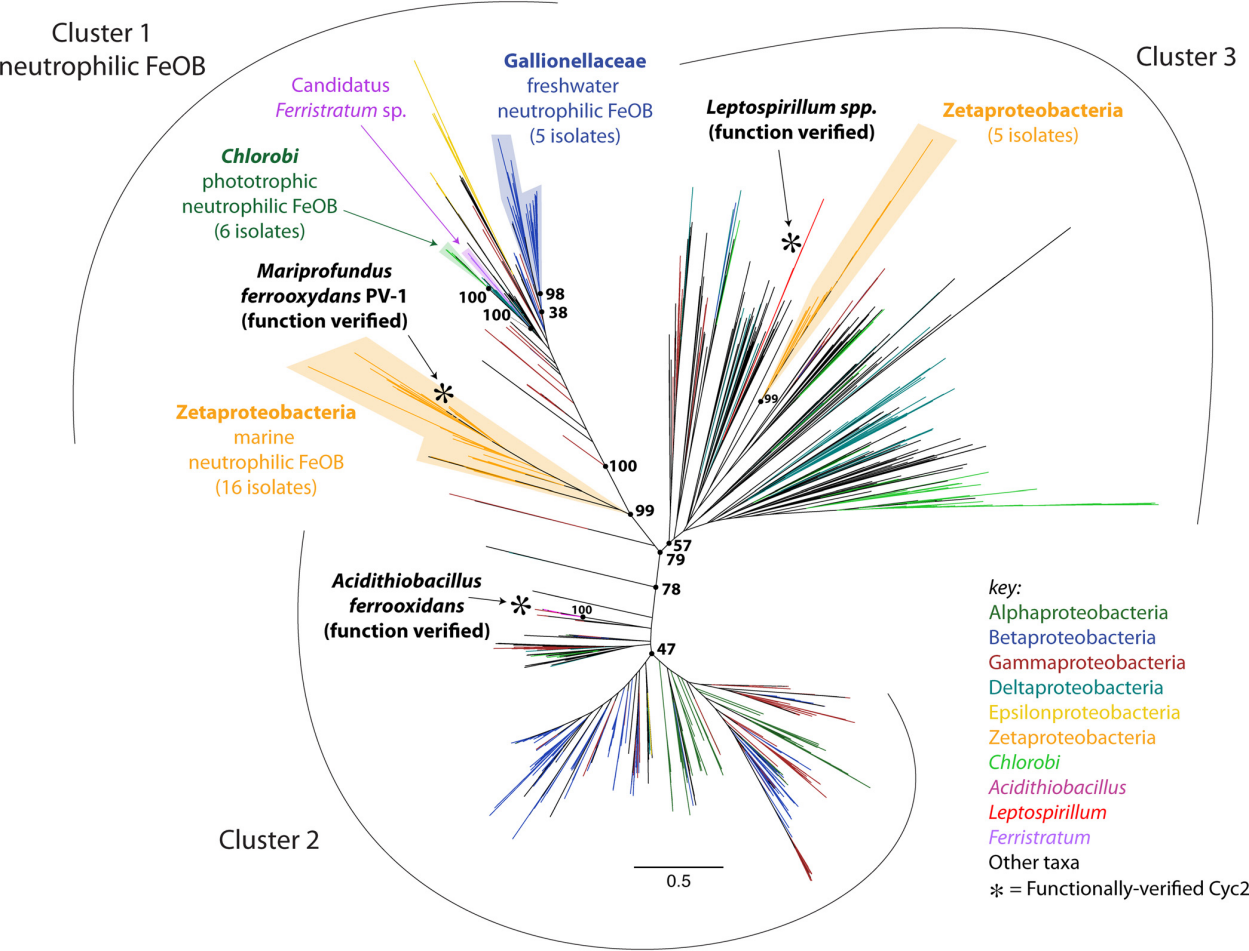
Updated Cyc2 maximum likelihood phylogenetic tree (1,593 sequences total). Sequences come from isolates, enrichments, single amplified genomes, and metagenome-assembled genomes, with few replicates. The three Cyc2 clusters continue to be supported (79% bootstrap support) and are confirmed with HMMs from FeGenie. Functionally characterized Cyc2 are shown with asterisks, with one from each cluster. Known neutrophilic Fe(II)-oxidizing clades are labeled with the number of distinct isolates represented by each. The full rectangular version of the tree with leaf labels is shown in figshare File 2 at https://doi.org/10.6084/m9.figshare.c.5390285.

In the short time since the first comprehensive Cyc2 phylogenetic tree was published (7), many more *cyc2* homologs have been sequenced. To explore how these new sequences fit into our understanding of Cyc2 phylogeny, we updated the Cyc2 tree with sequences from databases that met the criteria of our in-house pipeline—specifically a minimum length (365 amino acids), presence of a heme-binding site, and low blastp similarity cutoff (1E−5). Even with the substantial increase in Cyc2 homologs, the topology of the tree remained the same, with strong support for three main clusters of Cyc2 sequences (Fig. 9; see figshare File 2 at https://doi.org/10.6084/m9.figshare.c.5390285).

We have historically relied on our in-house pipeline for identifying Cyc2 homologs because the Cyc2 sequence is dominated by a porin domain that characteristically features low amino acid conservation (71). In contrast, the short cytochrome domain is the most conserved region (Fig. S6 and S7), and a consensus sequence derived from all homologs includes an AXPXFAR[Q/K][T/Y] motif located 5 amino acids upstream of the CXXCH heme-binding site, and a PXL motif 4 amino acids downstream of the CXXCH (Fig. S8). The PXL motif can be found in many other cytochromes, such as MtoD (72) and Cyc1 in *A. ferrooxidans* (CYC41 in reference 73); the proline and lysine appear to help stabilize the heme (73). In contrast, the AXPXFAR[Q/K][T/Y] motif is unique to Cyc2, and therefore can be used to identify Cyc2-like cytochromes.

10.1128/mBio.01074-21.6 FIG S6 Full alignment of Cyc2 from representative neutrophilic and acidophilic FeOB. The orange line indicates the conserved cytochrome region. Download FIG S6, PDF file, 1.9 MB. Copyright © 2021 Keffer et al. 2021 Keffer et al. https://creativecommons.org/licenses/by/4.0/ This content is distributed under the terms of the Creative Commons Attribution 4.0 International license.

10.1128/mBio.01074-21.7 FIG S7 Histograms of pairwise amino acid identity of the full-length Cyc2 sequences (A), porin portion (B), and cytochrome portion (*n* = 156) (C). The cytochrome portion is more highly conserved than the porin. (D) Amino acid identities (AAI) of full-length Cyc2 sequences from FeOB and Tenderia electrophaga. AAI to biochemically characterized Cyc2 are shown in bold type. Note that organisms from Cluster 1, e.g., neutrophilic FeOB *Zetaproteobacteria*, *Gallionellaceae*, and *Chlorobi*, are most similar to one another. Download FIG S7, PDF file, 0.2 MB. Copyright © 2021 Keffer et al. 2021 Keffer et al. https://creativecommons.org/licenses/by/4.0/ This content is distributed under the terms of the Creative Commons Attribution 4.0 International license.

10.1128/mBio.01074-21.8 FIG S8 Comparison of motifs found in the conserved cytochrome domain of Cyc2. The sequence logo labeled “All” is built from 1,593 homologs. Each of the cluster logos are built from all sequences in each cluster (334 in Cluster 1, 858 in Cluster 2, and 401 in Cluster 3). Download FIG S8, PDF file, 0.5 MB. Copyright © 2021 Keffer et al. 2021 Keffer et al. https://creativecommons.org/licenses/by/4.0/ This content is distributed under the terms of the Creative Commons Attribution 4.0 International license.

Recently, we developed hidden Markov models (HMMs) to identify Cyc2 sequences. These HMMs are part of the FeGenie program (74), which identifies and assigns putative Cyc2 homologs to one of the three phylogenetic clusters, depending on which HMM is the highest-scoring match. The HMMs were built from representative sequences of the phylogenetic tree presented by McAllister et al. (7), and each phylogenetic cluster is represented by a unique HMM. An alignment of sequences from each cluster show some differences in the consensus motif between clusters (Fig. S8). The FeGenie algorithm confirms the presence of a heme-binding motif and excludes sequences shorter than 365 amino acids, but users should confirm the presence of a porin in FeGenie-identified Cyc2 homologs using secondary structure prediction software (e.g., HHPRED). We confirmed the FeGenie Cyc2 HMM cluster classification matched the location of the sequences in the updated Cyc2 tree, with only one exception (figshare File 2 at https://doi.org/10.6084/m9.figshare.c.5390285). The Cyc2 HMMs have been validated and are the recommended tool for finding new Cyc2 homologs (74).

### Interpreting Cyc2 sequences: toward the discovery of new iron oxidizers.

We now have biochemical evidence for iron oxidation function by three diverse Cyc2 representatives that each fall within one of the three main clusters. It is tempting to now attribute iron oxidation function to the various Cyc2 homologs found in organisms across the tree (Fig. 9), as well as new homologs found using the HMM-based strategy above. However, the degree of confidence depends heavily on context, particularly how closely related the homolog is to a characterized Cyc2. New sequences can be added to the Cyc2 phylogenetic tree using the full alignment file (figshare Files 3 and 4 at https://doi.org/10.6084/m9.figshare.c.5390285). In some cases, the new sequence may fall within a well-supported clade of known FeOB with good evidence for Cyc2 function, which will give confidence in its predicted role in iron oxidation. However, the many long branches on the Cyc2 tree indicate a large degree of divergence, notably in Cluster 3. The functionally characterized *Leptospirillum* Cyt_572_ has an especially long branch, and it also has an unusual heme prosthetic group (75), both of which could mean that Cyt_572_ is not necessarily representative of Cluster 3. In all, the function of *cyc2* homologs within Cluster 3 may be considered most uncertain, requiring further verification.

In contrast, Cluster 1 has shorter branches, indicating that the sequences are more closely related to one another and to the biochemically tested Cyc2_PV-1_. In fact, many of the sequences (∼55%) within this cluster are from well-characterized neutrophilic FeOB, i.e., the *Gallionellaceae*, *Zetaproteobacteria*, or *Chlorobi*, including all known iron-oxidizing isolates of those taxa. In addition, previous work has helped build a case for prevalent Cluster 1 Cyc2 function. Cluster 1 FeOB *cyc2* are highly expressed in iron-oxidizing environments, including *Gallionellaceae cyc2* in the Rifle alluvial aquifer (5) and *Zetaproteobacteria cyc2* in marine iron mats in three hydrothermal systems (7). This hints at their importance in iron oxidation, which is further supported by expression that corresponds to iron-oxidizing conditions stimulated in microcosms (7). Building on this, here we show that a Cluster 1 Cyc2 oxidizes Fe(II) and has an appropriate redox potential. Altogether, this gives a higher degree of confidence in generalizing the iron oxidation function across Cluster 1 Cyc2.

The Cyc2 phylogenetic tree shows that Cyc2 sequences are very diverse, with many homologs that are distantly related to Cyc2_PV-1_, *A. ferrooxidans* Cyc2, and Cyt_572_, notably much of Clusters 2 and 3. Thus, there is still much work to be done to verify Cyc2 function in both known FeOB and organisms not known to oxidize iron. It is certainly possible that Cyc2 has additional functions, so its role in iron oxidation should be tested by genetic methods, though these have proven to be challenging for FeOB. However, the vast majority of *cyc2*/Cyc2 sequences come from genomes of uncultured organisms. In these cases, how can we test the function? We recently discovered Cluster 1 *cyc2* sequences in metagenome-assembled genomes of “*Candidatus* Ferristratum” spp. from the DTB120 phylum (Fig. 9), found in deeper portions of hydrothermal iron microbial mats inhabited by well-characterized *Zetaproteobacteria* FeOB. To test whether the new “*Ca.* Ferristratum” *cyc2* is connected to iron oxidation, we added Fe(II) to iron mat sample microcosms and analyzed a time course of transcriptomes. The expression of *cyc2* and the nitrate reduction gene *narG* from “*Ca.* Ferristratum” increased concurrently in response to Fe(II) addition, suggesting this group of organisms couples iron oxidation to nitrate reduction (10). This approach can guide the exploration of the diversity, distribution, and roles of iron-oxidizing bacteria in environmental systems. If many other Cyc2 homologs prove to be iron oxidases, microbial iron oxidation may be more widespread than we currently recognize.

## MATERIALS AND METHODS

### Structural modeling.

Signal peptides were predicted using SignalP (http://www.cbs.dtu.dk/services/SignalP/) (40), and secondary elements were predicted using PSIPRED (http://bioinf.cs.ucl.ac.uk/psipred/) (42, 43). For identification of structural homologs to Cyc2, we uploaded sequences to the HHpred tool (https://toolkit.tuebingen.mpg.de/tools/hhpred) and searched against the PDB_mmCIF70_14_Oct database, available as part of the Max Planck Institute Bioinformatics Toolkit (44, 45, 48). Structural modeling was carried out by HHpred/MODELLER (48, 51), I-TASSER (49), and Phyre2 (50). The structural models from all three platforms were found to be in close agreement.

### Cloning and heterologous expression of Cyc2_PV-1_.

The *cyc2* gene of Mariprofundus ferrooxydans PV-1 (NCBI:protein accession no. AKN78226) was codon optimized for expression in Escherichia coli and synthesized by Genscript (Piscataway, NJ, USA) with a C-terminal *Strep* tag II (WSHPQFEK) (76). The native PV-1 signal sequence was replaced with the signal sequence of an E. coli outer membrane porin, *ompA* (WP_063091005 amino acids 1 to 21). The *cyc2* gene was cloned into the EcoRI/HindIII sites of the pMal-p4X plasmid (with the *malE* gene removed). This *cyc2*-containing plasmid was cotransformed into E. coli C41(DE3) (Lucigen) with pEC86*, a plasmid containing the cytochrome *c* maturation (*ccmABCDEFGH*) genes under a constitutive promoter, to ensure heme insertion into Cyc2_PV-1_ (77). The pEC86 used here (pEC86*) was a spontaneous mutant found to possess a frameshift and early stop codon in the *ccmA* gene. For unknown reasons that are beyond the scope of this article, this plasmid was not detrimental to yield and beneficial to detection of Cyc2_PV-1_ in our system, and was used in all expression studies.

The porin-only construct was created by removing the bases corresponding to the cytochrome domain from the plasmid via site-directed mutagenesis. The 8×His tag, TEV protease cleavage site, and linker region were inserted into the relevant constructs also by site-directed mutagenesis (NEB Q5-based kit). Optimized DNA sequence is provided in figshare File 1 (https://doi.org/10.6084/m9.figshare.c.5390285). Plasmids were propagated in E. coli NEB5α competent cells, and clones were sequenced after manipulation by Sanger sequencing at the University of Delaware Sequencing and Genotyping Center prior to being transformed into E. coli C41(DE3)/pEC86*.

For expression of *cyc2*, E. coli was grown aerobically at 37°C (with shaking at 200 rpm) in lysogeny broth (LB), with ampicillin (100 μg/ml) for propagation of the pMal-p4X-based plasmids, and chloramphenicol (30 μg/ml) for the pEC86* plasmid. LB was routinely supplemented with 2 mM MgCl_2_, trace vitamins and minerals (78), and ferric citrate. After the cultures reached an optical density at 600 nm (OD_600_) of ∼0.5, they were induced with 0.1 mM isopropyl-β-d-1-thiogalactopyranoside (IPTG) for derepression of the *lac* operon; induction proceeded for 3 to 4 h at 30°C with shaking at ∼200 rpm. We evaluated the use of 5-aminolevulic acid for improving expression (79, 80) but found no benefit.

### Purification of Cyc2_PV-1_ and assays.

Cells were harvested by centrifugation (6,000 × *g*) for 15 min at 4°C, and the pellets were stored at −20°C until lysed. Cells were lysed by a passage through the Avestin C5 Emulsiflex homogenizer at 10,000 to 15,000 lb/in^2^ in 20 mM Tris (pH 8) with protease inhibitors and 10 μg/ml DNase. After lysis, cellular debris was removed by centrifugation at 15,000 × *g* for 20 min at 4°C. Total membranes were harvested by ultracentrifugation at 100,000 × *g* for 1 h at 4°C in a Beckman Coulter SW28 or SW32 rotor. Total membranes were resuspended in 20 mM Tris (pH 8)−1.5 M sucrose−1% *n*-dodecyl β-d-maltoside (DDM). The membranes were solubilized by rotating at 4°C overnight. Solubilized membranes were mixed 1:1 with binding buffer (20 mM Tris [pH 8]−1 M NaCl−20 mM imidazole) and loaded onto a HisPur nickel-nitrilotriacetic acid (Ni-NTA) (Thermo Scientific) column equilibrated with 20 mM Tris (pH 8)−750 mM sucrose−500 mM NaCl−10 mM imidazole−0.2% DDM (equilibration buffer). The column flowthrough was collected, and the column was washed with 10 column volumes of equilibration buffer. After the column was washed, bound protein was eluted with 300 mM imidazole in equilibration buffer.

For the redox assays, eluted protein containing Cyc2_PV-1_ was dialyzed against equilibration buffer to remove the high concentration of imidazole. Then the His tag was cleaved by incubation with TEV protease (NEB) for 18 h at room temperature. Following cleavage, the fraction was reloaded onto an equilibrated Ni-NTA column, and tag-free Cyc2_PV-1_ was not retained by the column while TEV protease was. The tag-free Cyc2_PV-1_ was dialyzed against assay buffer (20 mM morpholineethanesulfonic acid [MES] [pH 6.3]−300 mM NaCl−500 mM sucrose−0.1% DDM) to adjust the buffer conditions for the assay. After dialysis, the protein was concentrated using a 30-kDa molecular weight cutoff (MWCO) spin concentrator (Amicon Ultra). Cyc2_PV-1_ was degassed and taken into the anaerobic chamber (Coy 5% H_2_−95% N_2_ atmosphere) and diluted into anoxic assay buffer. Diluted Cyc2_PV-1_ was sealed in a masked, quartz microcuvette with a septum lid (Starna Cells) and then removed from the chamber for UV-Vis readings on a NanoDrop OneC spectrophotometer (Thermo Scientific). The microcuvette was returned to the anaerobic chamber, and 2 mM ferrous citrate was added to the cuvette, before it was resealed and taken back out to the spectrophotometer. Spectra from 200 to 800 nm were collected every min for ∼30 min. The reduced Cyc2_PV-1_ spectra (Fig. 5A) were obtained by adding a final concentration of 5 mM sodium dithionite to Cyc2_PV-1_ in assay buffer. The oxidized Cyc2_PV-1_ spectra (Fig. 5A) were obtained by adding a final concentration of 100 μM potassium hexacyanoferrate(III) to Cyc2_PV-1_ in assay buffer.

The redox potential was measured by a modified Massey method (63–65). Cyc2_PV-1_ was purified as described above, then concentrated, and buffer exchanged into 20 mM Tris (pH 8)−300 mM NaCl−300 mM sucrose−0.02% DDM using the Amicon spin concentrator. Cyc2_PV-1_ was degassed and taken into the anaerobic chamber and diluted into anoxic assay buffer. Diluted Cyc2_PV-1_ was sealed in a masked, quartz microcuvette with a septum lid and then removed from the chamber for UV-Vis readings on an Agilent Cary3500 spectrophotometer. Cyc2_PV-1_ was taken back in the chamber for addition of the reference dye, 2,6-dichlorophenolindophenol (DCPIP) (redox potential *E*_m_ = 217 mV) and 500 μM xanthine (both from Sigma-Aldrich), then sealed and removed to take UV-Vis readings on the spectrophotometer. The reaction was initiated at the spectrophotometer by adding anoxic xanthine oxidase (microbial; 1 to 3 μg/ml; Sigma-Aldrich) via syringe and needle through the septum lid. Readings of 350 to 800 nm were collected every 15 s for ∼1 h. The absorbance change for the reduced Cyc2_PV-1_ Soret maximum was monitored at 429 nm, at which there was negligible contribution from DCPIP, and the absorbance change for the dye peak was monitored at 610 nm, at which there was negligible contribution from Cyc2_PV-1_. These changes in absorbance were transformed by the Nernst equation and plotted: the one-electron reduction of Cyc2_PV-1_ with 25 mV ln (Cyc2_red_/Cyc2_ox_) (where Cyc2_red_ is reduced Cyc2 and Cyc2_ox_ is oxidized Cyc2) and the two-electron reduction of DCPIP with 12.5 mV ln (DCPIP_red_/DCPIP_ox_). Data points where the reduced/oxidized ratio was greater than 10 or less than 0.065 were excluded from the analysis. The *y* intercept of a line fit to the data represents the difference in potential between the heme and the known potential of DCPIP. The reduction potential of Cyc2_PV-1_ was calculated based on four independent titrations. Bovine heart cytochrome *c* (Sigma-Aldrich) was used as a positive control to optimize experimental conditions.

### SDS-PAGE, Western blotting, and heme staining.

Whole cells to be analyzed by sodium dodecyl sulfate-polyacrylamide gel electrophoresis (SDS-PAGE) were lysed by resuspension of a cell pellet in 5× SDS running buffer (125 mM Tris, 1.25 M glycine, 0.5% SDS [pH 8.3]) and passing the resuspension through a 27.5-gauge needle 10 times. Samples were incubated 1:1 in 2× loading buffer (250 mM Tris, 2% SDS, 30% glycerol, 0.002% bromophenol blue) prior to loading on a 15% Tris-buffered polyacrylamide resolving gel, topped with a 5% polyacrylamide stacking gel, and electrophoresed according to the method of Laemmli (81). Samples were electrophoresed at 70 V for 30 to 45 min and then at 125 V for 45 to 60 min. The gel was then stained with either Coomassie blue (1 g Coomassie brilliant blue stain in 10% acetic acid−40% ethanol) or o-dianisidine for heme. For heme staining (82), 0.1 g *o*-dianisidine was dissolved in 90 ml water. Immediately prior to the start of staining, 10 ml sodium citrate (pH 4.4) and 200 μl hydrogen peroxide were added to the *o*-dianisidine solution. Heme-containing proteins in the gel turned brown after 15 min to 2 h of incubation in the heme stain at room temperature. For Western blotting and another version of the heme stain, the proteins from the SDS-polyacrylamide gel were transferred to a polyvinylidene difluoride (PVDF) membrane at 30 V for 16 h (4°C) in transfer buffer (25 mM Tris, 192 mM glycine, 20% methanol [pH 7.2]). Heme peroxidase activity was assessed by washing the membrane with TBST buffer (20 mM Tris, 137 mM NaCl, 0.1% Tween 20 [pH 7.6]), and incubating for 15 to 30 min with Pierce ECL luminol and substrate (83), before imaging on a Typhoon FLA 9500 (GE Healthcare Life Sciences) or iBright FL1500 system (Invitrogen). For *Strep* tag II detection, the PVDF membrane was blocked for 1 h with 1% bovine serum albumin (BSA) in TBST buffer, rinsed with TBST, and incubated for 1 h with Precision Protein Streptactin-HRP (horseradish peroxidase) conjugate (1:60,000 dilution; Bio-Rad). The membrane was then washed with four 5-min washes in TBST and then incubated with Pierce ECL luminol and substrate for 5 min before imaging on the Typhoon FLA 9500 or iBright system.

### Separation of inner and outer membrane layers.

Cyc2_PV-1_ was expressed in E. coli C41(DE3)/pEC86* as described above, and cells were lysed on the Emulsiflex as described above. After lysis, cellular debris was removed by centrifugation at 15,000 × *g* for 20 min at 4°C. A sucrose gradient was set up with 7 ml of 1.5 M sucrose as the bottom layer, 7 ml of 0.5 M sucrose as the middle layer, and 15 ml of lysed cells as the top layer (58, 59). The gradient was ultracentrifuged at 100,000 × *g* for 18 h at 4°C in a Beckman Coulter SW28 or SW32 rotor. Following ultracentrifugation, the outer membrane could be found at the bottom of the tube, and the inner membrane localized to the interface between the 1.5 M and 0.5 M sucrose layers. Cytoplasmic proteins remained above the 0.5 M sucrose layer.

### Mass spectrometry. (i) Sample preparation.

Gel bands of interest were excised for analysis. The enzymatic digestion procedure was performed with trypsin (Promega) at 37°C as previously described (84) and included reduction/alkylation with dithiothreitol (Bio-Rad) and iodoacetamide (Sigma), respectively. Subsequently, samples were desalted and concentrated using μC18 Ziptips (Millipore) per the manufacturer’s instructions, then dried with a SpeedVac vacuum concentrator (Thermo Fisher), and resuspended in 24 μl of 2% acetonitrile (ACN), 0.1% formic acid (FA).

### (ii) LC-MS/MS data acquisition.

Liquid chromatography-tandem mass spectrometry (LC-MS/MS) was performed on a TripleTOF 6600 (Sciex) coupled to an Eksigent nanoLC 425 (Sciex) operating in microLC flow mode. Twelve microliters of each sample was injected onto a ChromXP C18CL column (3 μm, 120 Å, 150 mm × 0.3 mm; Sciex). Gradient elution was performed with mobile phase A (0.1% formic acid in water; Fisher) and mobile phase B (0.1% formic acid in ACN; Fisher) at a flow rate of 5 μl/min. A program of 3% mobile phase B to 35% mobile phase B in 5 min, 35% mobile phase B to 80% mobile phase B in 1 min, and 80% mobile phase B for 2 min was used to elute peptides. The eluate was ionized with a dual spray source. The mass spectrometer was operated in positive ion mode with a full MS1 scan experiment in a mass range of 400 to 1,250 *m/z* with a scan time of 250 ms followed by MS/MS experiments in the mass range of 100 to 1,500 *m/z* with a scan time of 50 ms. The top 30 precursor ions were selected for fragmentation.

### (iii) Database search.

LC-MS/MS data were searched against a database of all E. coli K-12 protein sequences (available at ftp.ncbi.nlm.nih.gov), modified to include Cyc2_PV-1_, with ProteinPilot v5.1 (Sciex) using the Paragon algorithm. Search parameters were specified as following: iodoacetamide cysteine modification, trypsin enzyme, gel identifier (ID) special factors. Protein identifications with a local false discovery rate (FDR) of 1%, and at least two high-confidence unique peptides (99% confidence) were considered.

### Cyc2 phylogeny and amino acid identity calculations.

The Cyc2 phylogenetic tree was constructed following the same methods as published previously (7). The database of 634 full-length dereplicated Cyc2 sequences was updated by adding new sequences identified by blastp (85) against the NCBI and IMG databases (maximum E value of 1 × 10^−5^) using one query from each of the three Cyc2 clusters. Resulting sequences were dereplicated using cd-hit at 100% identity over 90% of the length of the protein (86). The database was further screened to remove sequences either shorter than 365 amino acids or lacking the cytochrome *c*-binding motif (CXXCH) or the upstream PXFAR[Q/K][T/Y] motif and several sequences from highly sampled genera (*Burkholderia*, *Ralstonia*, and *Xanthomonas*). This resulted in a total of 1,593 full-length sequences. To generate the tree, these sequences were aligned using MUSCLE (87) (figshare File 3 at https://doi.org/10.6084/m9.figshare.c.5390285), the alignment was masked to remove positions with greater than 30% gaps (figshare File 4 at https://doi.org/10.6084/m9.figshare.c.5390285), and a maximum likelihood phylogenetic tree was built using RAxML (276 alignment columns, 300 bootstraps, CAT model of rate heterogeneity, JTT amino acid substitution model) (88). The resulting phylogenetic tree was colored, and names were customized using the Iroki program (89). Cyc2 cluster assignments were confirmed by running all sequences through the FeGenie program (74). Amino acid identity (AAI) values were calculated from the pairwise alignments prior to masking.

### HMM development and calibration.

Representative sequences from each Cyc2 phylogenetic cluster were aligned using MUSCLE (87) and then manually curated. These alignments were then used to construct hidden Markov models (HMMs) with HMMER (90). The HMMs were calibrated by querying each HMM against NCBI’s nonredundant (nr) protein database. The results of each search were visually inspected to identify the optimum bit score cutoff for each HMM. Due to the poor conservation of the porin domain of Cyc2, many Cyc2 homologs were identified at low bit scores; these low-scoring homologs were confirmed by (i) visual inspection of the sequence at the N terminus to confirm the presence of the conserved cytochrome motif and (ii) secondary structure modeling using HHPRED (48). Occasionally, false positives were identified at similar bit score values to many true positives (e.g., porins without heme-binding motifs). To exclude these types of false positives, any protein matching a Cyc2 HMM without a heme-binding motif is automatically excluded from the results through a built-in heme-detecting function. Additionally, because the vast majority of confirmed Cyc2 homologs are at least 400 residues in length, FeGenie is programmed to exclude any match to the Cyc2 HMMs shorter than 365 residues.

### Additional supplemental material.

Additional supplemental material files are available at https://doi.org/10.6084/m9.figshare.c.5390285.
