# Supplementary material for: Iron Oxidation by a Fused Cytochrome-Porin Common to Diverse Iron-Oxidizing Bacteria
Source: mBio. 2021 Jul 27;12(4):e01074-21. doi: 10.1128/mBio.01074-21 (PMC8406198; doi:10.1128/mBio.01074-21)
Supplement: FIG S2 [file mbio.01074-21-sf002.pdf]

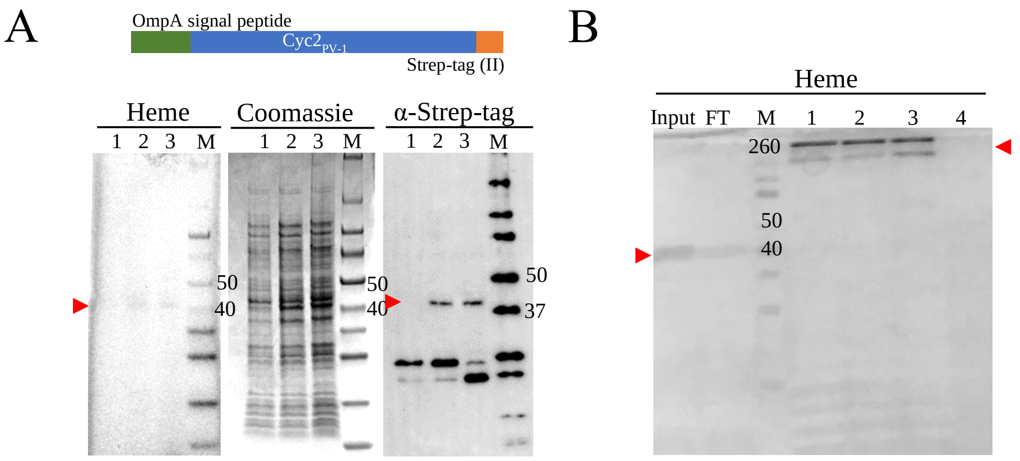

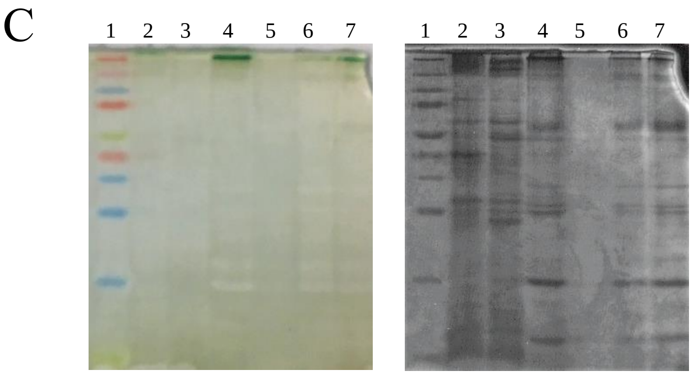

1. Spectra Broad Range protein ladder
2. insoluble total membrane pellet
3. Purification flow-through
4. Purification elution fraction
5. Post His-tag cleavage
6. Tag-free fraction
7. Fraction concentrated and used in assay

Figure S2. Constructs and expression of Cyc2$_{\text{PV-1}}$. Cyc2$_{\text{PV-1}}$ is marked with a red arrowhead, protein ladder is labeled with an M (Spectra Broad Range on heme and Coomassie, WesternC on α-*Strep*-tag Western blot), and relevant band sizes are labeled in kDa. (A) Schematic of gene construct for expression and representative stained SDS-PAGE gels showing Cyc2$_{\text{PV-1}}$ expression in *E. coli*: 1) uninduced, 2) induced, 3) lysed and induced. Smaller bands visible on *Strep*-tag Western blots are non-specific. (B) Heme-stained gel of fractions during His-tag purification. Cyc2$_{\text{PV-1}}$ migrates at its expected molecular weight in the diluted total membranes (input) and flow-though (FT). After elution, Cyc2$_{\text{PV-1}}$ migrates in a high-molecular weight complex (1 - 300 μM imidazole, 2 - after dialysis to remove imidazole). After TEV protease cleavage of the His-tag, Cyc2$_{\text{PV-1}}$ does not interact with the Ni-NTA column (3 - flow-through, 4 - imidazole elution). (C) Uncropped gel corresponding to Figure 2D. See lane labels to the right of image.
