# Supplementary material for: Iron Oxidation by a Fused Cytochrome-Porin Common to Diverse Iron-Oxidizing Bacteria
Source: mBio. 2021 Jul 27;12(4):e01074-21. doi: 10.1128/mBio.01074-21 (PMC8406198; doi:10.1128/mBio.01074-21)
Supplement: TABLE S1 [file mbio.01074-21-st001.pdf]

Table S1. Unique peptides detected by tandem MS/MS that matched to Cyc2$_{PV-1}$ with 99% confidence

**Sequence**	**ΔMass**	**Obs. MW**	**Theor. MW**	**Modi-fications**
ADLKPLHNLGVGIGYAYQK	3.24E-03	2056.114	2056.111	
ADLKPLHNLGVGIGYAYQKQTPGAAAAAGSVAQK	-2.54E-03	3364.787	3364.789	
FDAWSIR	2.21E-03	893.4417	893.4396	
GKTSTDPAVVGGNFYGAAGLNTAGAK	5.05E-03	2423.213	2423.208	
GVLTTELGGFDTLVGFGYVTGDAGR	-0.00189	2501.242	2501.244	
ITTAQIAAFYEIYQNFEINLIYNSAK	4.00E-03	3037.547	3037.544	
MGSFTDVGEQALVEDDNLSLPAVLNATVVIR	-1.31E-03	3272.657	3272.66	
PTTVVAPTVPGVGPVPM	-5.37E-04	1617.88	1617.88	
QTGAACLSCHFQTFPALNAFGR	-1.99E-04	2453.137	2453.137	*a*
QTPGAAAAAGSVAQK	0.000334	1309.663	1309.663	*b*
QTPGAAAAAGSVAQKITTAQIAAFYEIYQNFEINLIYNSAK	-3.02E-03	4346.22	4346.222	
TSTDPAVVGGNFYGAAGLNTAGAK	-0.00012	2238.092	2238.092	
TTGVASTGTWNVPSETPILIAGR	-0.00015	2331.207	2331.207	*c*
TVNGGLVAGANTTTNTTTIEFEGLLWSHPQFEK	-0.00022	3532.748	3532.747	
VGVSAHNSTFGGSAIMEYSNVFGQHSGK	-0.00157	2867.328	2867.33	

[a]Carbamidomethyl at C6 and C9

[b]Gln->pyro-Glu at N-term

[c]Trp->Kynurenin at W10
