# Supplementary material for: Iron Oxidation by a Fused Cytochrome-Porin Common to Diverse Iron-Oxidizing Bacteria
Source: mBio. 2021 Jul 27;12(4):e01074-21. doi: 10.1128/mBio.01074-21 (PMC8406198; doi:10.1128/mBio.01074-21)
Supplement: FIG S3 [file mbio.01074-21-sf003.pdf]

A

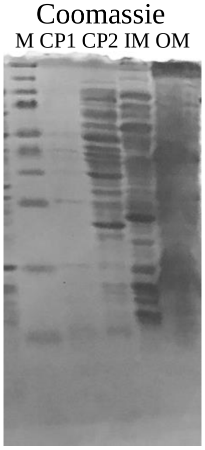

B

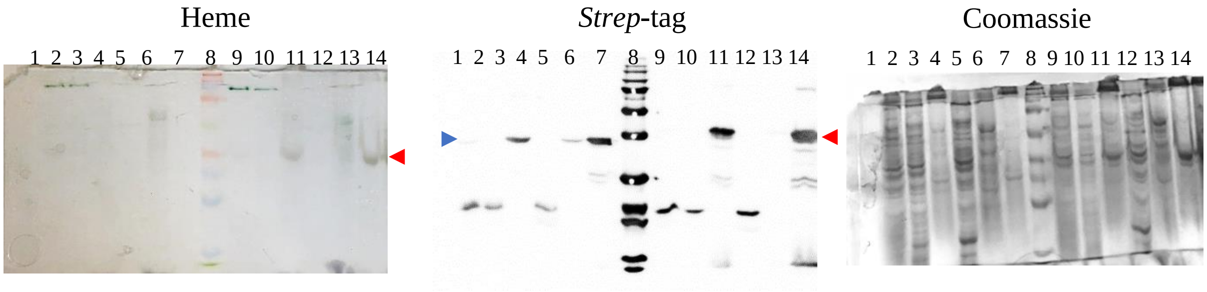

Figure S3. Uncropped gels. (A) Coomassie-stained gel corresponding to Fig. 3A. CP1, CP2 - cytoplasmic proteins, IM - inner membranes, OM - outer membranes, M - Spectra Broad Range protein ladder with relevant bands in kDa. (B) Gels corresponding to samples in Fig. 4. Red arrow indicates full-length $Cyc2_{PV-1}$ and blue arrow indicates porin-only. Lanes: 1-empty vector, 2-porin lysed supernatant, 3-porin ultracentrifuged supernatant, 4-porin total membranes, 5-porin cytoplasmic proteins, 6-porin inner membranes, 7-porin outer membranes, 8-Spectra Broad Range or WesternC protein ladder, 9- $Cyc2_{PV-1}$ lysed supernatant, 10- $Cyc2_{PV-1}$ ultracentrifuged supernatant, 11- $Cyc2_{PV-1}$ total membranes, 12- $Cyc2_{PV-1}$ cytoplasmic proteins, 13- $Cyc2_{PV-1}$ inner membranes, 14- $Cyc2_{PV-1}$ outer membranes.
