# Supplementary material for: Iron Oxidation by a Fused Cytochrome-Porin Common to Diverse Iron-Oxidizing Bacteria
Source: mBio. 2021 Jul 27;12(4):e01074-21. doi: 10.1128/mBio.01074-21 (PMC8406198; doi:10.1128/mBio.01074-21)
Supplement: TABLE S2 [file mbio.01074-21-st002.pdf]

Table S2. Relevant Fe(II) and citrate speciation in the iron oxidase assay buffer[a] from Visual MINTEQ calculation (https://vminteq.lwr.kth.se/).

Component	% of total concentration	Species name
Citrate^{3-}	9.559	Citrate^{3-}
	0.505	FeH-citrate (aq.)
	10.261	Na-citrate^{2-}
	2.539	H-citrate^{2-}
	0.029	H2-citrate^{-}
	77.107	Fe-citrate^{-}
Fe^{2+}	21.282	Fe^{2+}
	0.505	FeH-citrate (aq.)
	10.99	$FeCl^{+}$
	77.107	Fe-citrate^{-}
Na^{+}	93.288	Na^{+}
	0.068	Na-citrate^{2-}
	6.644	NaCl (aq.)
Cl^{-}	93.393	Cl^{-}
	6.6	NaCl (aq.)
MES^{-}	60.951	MES^{-}
	39.049	H-MES (aq.)

[a]Sucrose and DDM were in assay buffer but not available as part of the calculation
