# Supplementary figures and images for: Iron Oxidation by a Fused Cytochrome-Porin Common to Diverse Iron-Oxidizing Bacteria
Source: mBio. 2021 Jul 27;12(4):e01074-21. doi: 10.1128/mBio.01074-21 (PMC8406198; doi:10.1128/mBio.01074-21)

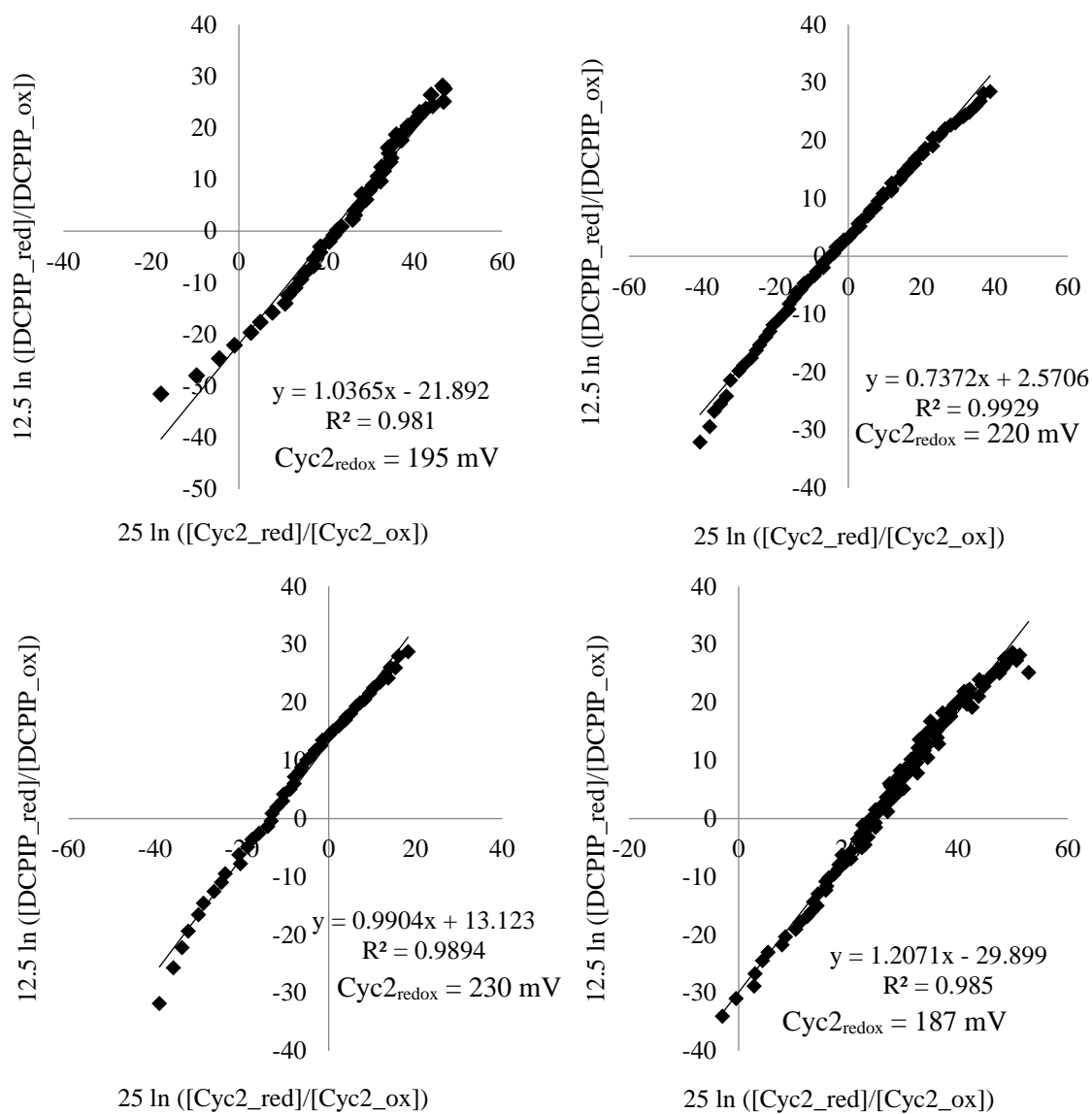

**Figure S4.** Four independent redox titration reactions with Cyc2<sub>PV-1</sub> and DCPIP.

Supplement: FIG S4 [file mbio.01074-21-sf004.pdf]
