# Supplementary material for: Iron Oxidation by a Fused Cytochrome-Porin Common to Diverse Iron-Oxidizing Bacteria
Source: mBio. 2021 Jul 27;12(4):e01074-21. doi: 10.1128/mBio.01074-21 (PMC8406198; doi:10.1128/mBio.01074-21)
Supplement: FIG S5 [file mbio.01074-21-sf005.pdf]

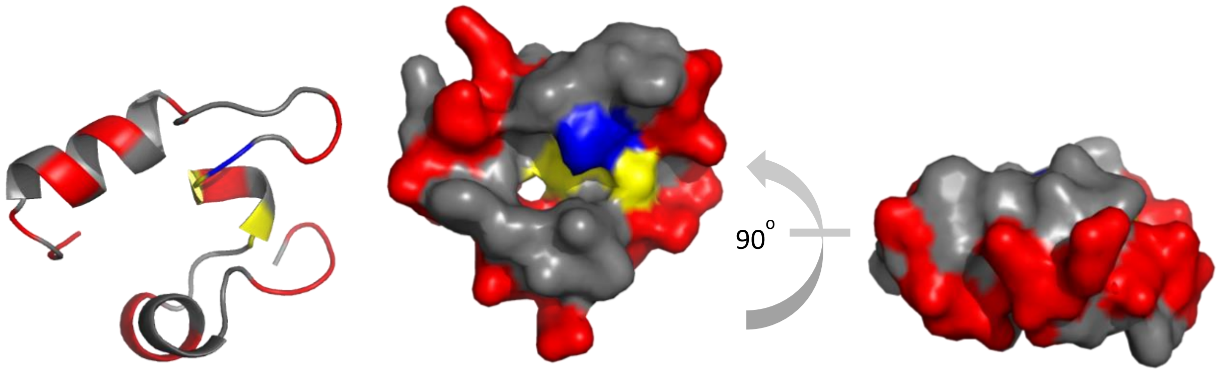

Figure S5. Three views of the modeled cytochrome domain of Cyc2$_{PV-1}$. The view on the right is rotated 90 ^{o} away from the viewer compared to the view in the center. Hydrophobic residues are gray and polar residues are red. Heme (not pictured) is covalently attached to cysteine residues (yellow) and coordinated by histidine (blue). Model generated using MODELLER (B. Webb, A. Sali, Curr Prot Bioinf, 54: 5.6.1-5.6.37, 2016, https://doi: 10.1002/cpbi.3).
