# Supplementary material for: Iron Oxidation by a Fused Cytochrome-Porin Common to Diverse Iron-Oxidizing Bacteria
Source: mBio. 2021 Jul 27;12(4):e01074-21. doi: 10.1128/mBio.01074-21 (PMC8406198; doi:10.1128/mBio.01074-21)
Supplement: FIG S6 [file mbio.01074-21-sf006.pdf]

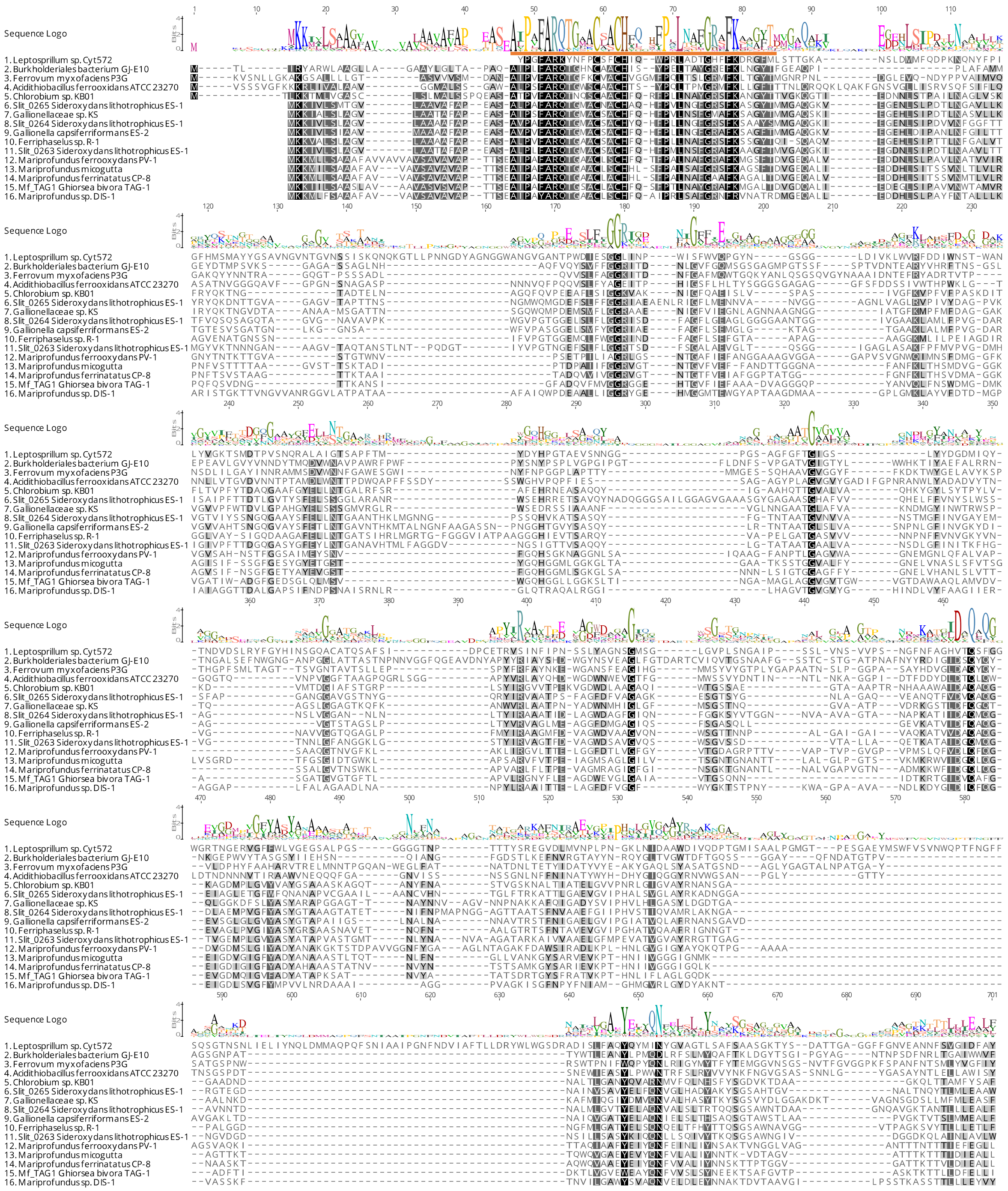

Figure S6. Full alignment of Cyc2 from representative neutrophilic and acidophilic FeOB. Orange line indicates the conserved cytochrome region.
