# Supplementary material for: Iron Oxidation by a Fused Cytochrome-Porin Common to Diverse Iron-Oxidizing Bacteria
Source: mBio. 2021 Jul 27;12(4):e01074-21. doi: 10.1128/mBio.01074-21 (PMC8406198; doi:10.1128/mBio.01074-21)
Supplement: FIG S7 [file mbio.01074-21-sf007.pdf]

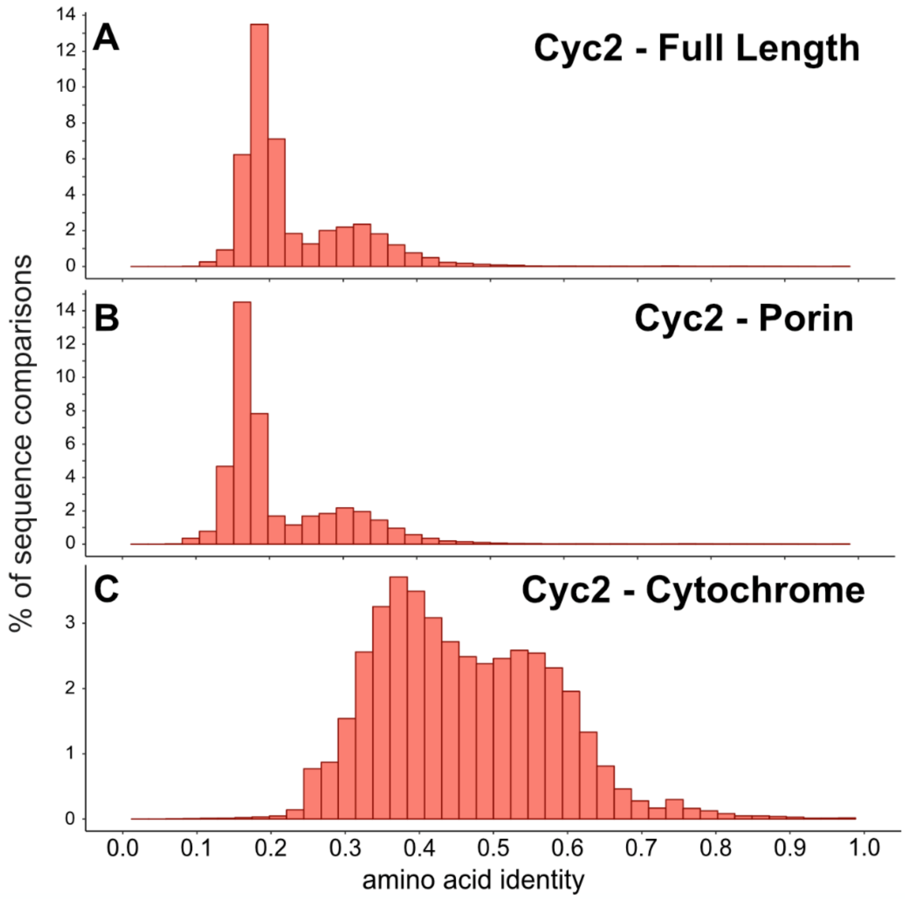

		Cluster 1									Cluster 2		Cluster 3			
		Zetaproteobacteria				Gallionellaceae				Chlorob.	**Lepto**	Tenderia	Ferrovum	Dechlor.	Burkh.	**Acidith.**
Cluster	Taxa	TAG-1	DIS-1	PV-1	CP-8	KS	ES-1	ES-2	OYT1	luteo.	**ferrodz.**	electro.	myxo.	RCB	GJ-E10	**ferrox.**
1	Ghiorsea bivora TAG-1															
	Mariprofundus sp. DIS-1	29.9%														
	M. ferrooxydans PV-1	38.9%	33.5%													
	M. ferrinatatus CP-8	47.8%	33.1%	45.7%												
	Gallionellaceae sp. KS	28.2%	26.9%	30.1%	28.3%											
	Sideroxydans lithotrophicus ES-1	29.2%	27.3%	29.1%	29.1%	39.6%										
	Gallionella capsiferriformans ES-2	30.4%	26.4%	29.5%	30.6%	36.8%	53.4%									
	Ferriphaselus amnicola OYT1	29.9%	28.8%	29.6%	28.1%	34.2%	51.1%	52.2%								
	Chlorobium luteolum	25.1%	26.2%	28.3%	26.3%	35.2%	33.5%	32.7%	33.5%							
2	**Leptospirillum ferrodiazotrophum**	**18.8%**	**17.5%**	**16.4%**	**15.2%**	**19.4%**	**19.8%**	**18.1%**	**16.5%**	**17.9%**						
	Tenderia electrophaga	19.0%	17.5%	21.5%	18.5%	19.7%	19.2%	17.6%	17.8%	20.4%	**17.5%**					
3	Ferrovum myxofaciens P3G	19.9%	19.4%	22.0%	19.5%	22.1%	22.6%	20.1%	19.6%	19.6%	**19.6%**	18.9%				
	Dechloromonas aromatica RCB	21.6%	17.2%	19.7%	19.3%	18.6%	19.5%	19.5%	19.1%	18.2%	**18.2%**	17.8%	26.8%			
	Burkholderiales bacterium GJ-E10	21.1%	19.6%	19.8%	19.6%	20.0%	22.1%	22.7%	21.7%	19.7%	**17.4%**	17.8%	30.5%	27.3%		
	**Acidithiobacillus ferrooxidans ATCC2327**	**18.0%**	**21.5%**	**19.6%**	**18.9%**	**22.0%**	**23.0%**	**21.3%**	**23.1%**	**20.0%**	**18.6%**	**19.3%**	**28.1%**	**26.7%**	**28.2%**	
	Thiomonas sp. FB-6	22.0%	20.9%	21.8%	20.6%	22.7%	19.4%	20.4%	22.9%	21.4%	**17.4%**	19.1%	29.2%	35.2%	30.5%	**29.6%**

Figure S7. Histograms of pairwise amino acid identity of the (A) full length Cyc2 sequences, (B) porin portion, and (C) cytochrome portion (n=156). The cytochrome portion is more highly conserved than the porin. (D) Amino acid identities (AAI) of full length Cyc2 sequences from FeOB and *Tenderia electrophaga*. AAI to biochemically characterized Cyc2 are shown in bold. Note that organisms from “Cluster 1” e.g. neutrophilic FeOB Zetaproteobacteria, Gallionellaceae, and *Chlorobi* are most similar to one another.
