# Supplementary material for: Iron Oxidation by a Fused Cytochrome-Porin Common to Diverse Iron-Oxidizing Bacteria
Source: mBio. 2021 Jul 27;12(4):e01074-21. doi: 10.1128/mBio.01074-21 (PMC8406198; doi:10.1128/mBio.01074-21)
Supplement: FIG S8 [file mbio.01074-21-sf008.pdf]

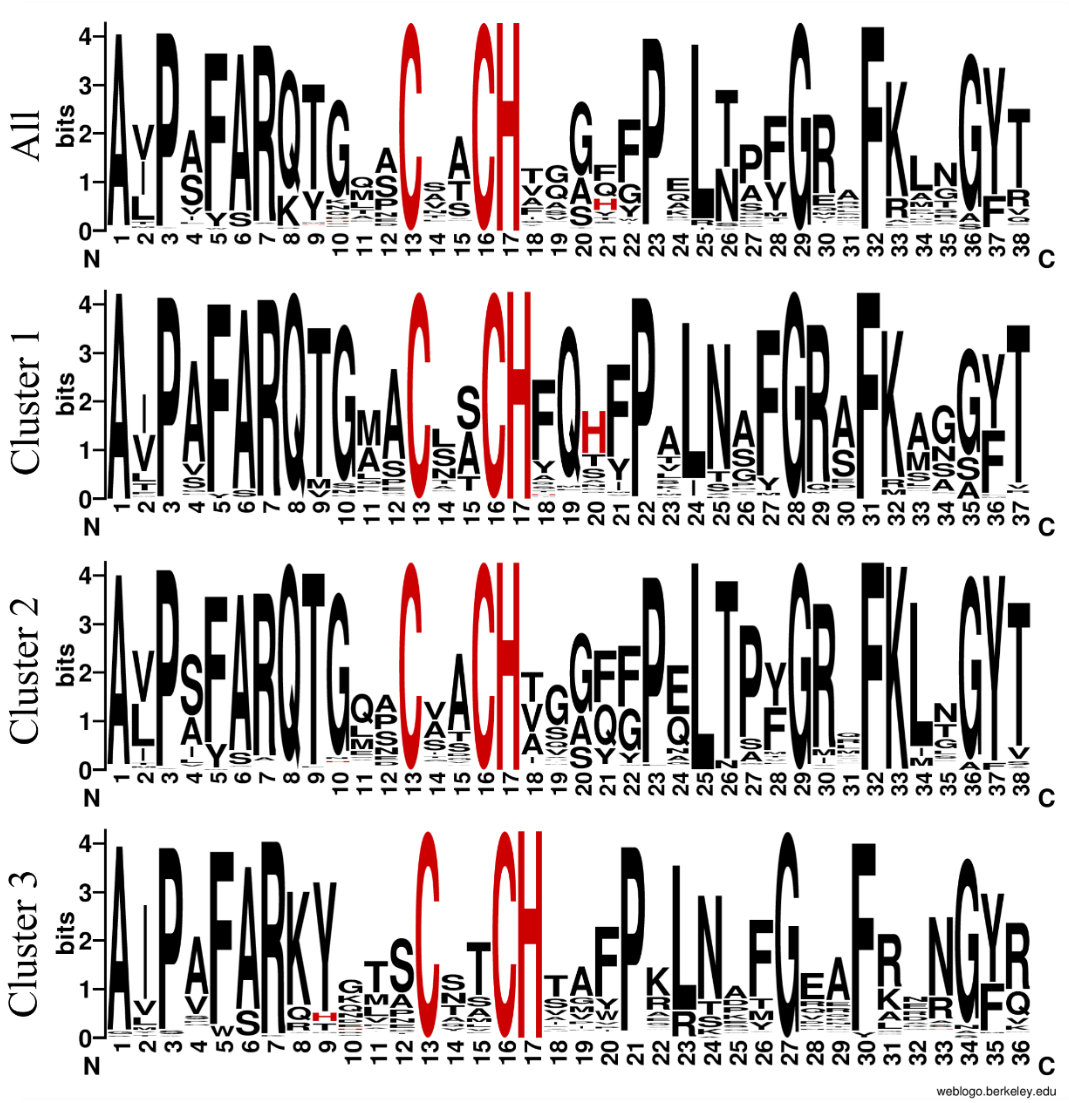

Figure S8. Comparison of motifs found in the conserved cytochrome domain of Cyc2. The sequence logo labeled “All” is built from 1593 homologs. Each of the Cluster logos are built from all sequences in each cluster (334 in Cluster 1, 858 in Cluster 2, and 401 in Cluster 3).
