# Supplementary material for: Iron Oxidation by a Fused Cytochrome-Porin Common to Diverse Iron-Oxidizing Bacteria
Source: mBio. 2021 Jul 27;12(4):e01074-21. doi: 10.1128/mBio.01074-21 (PMC8406198; doi:10.1128/mBio.01074-21)
Supplement: FIG S1 [file mbio.01074-21-sf001.pdf]

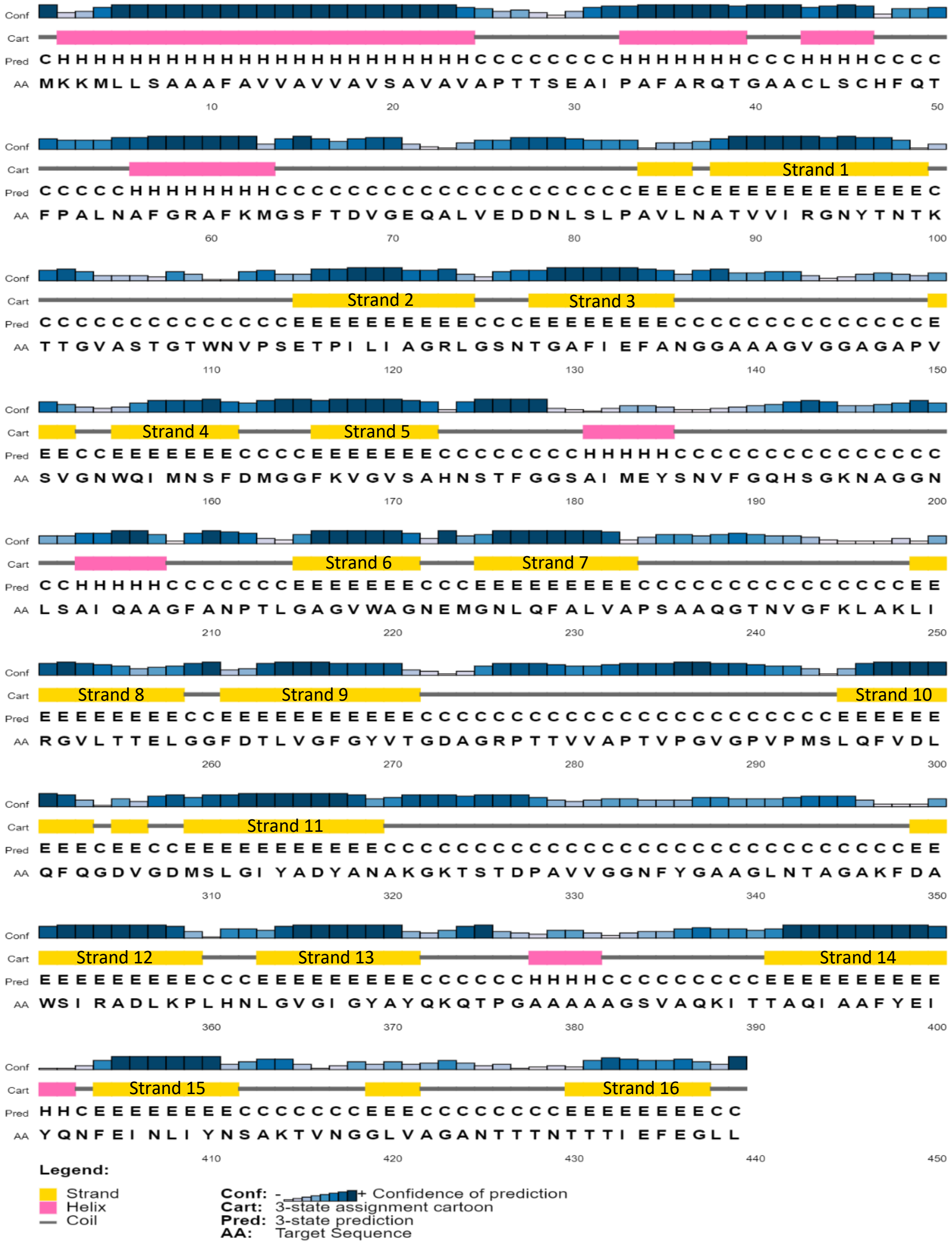

Figure S1. PSIPRED prediction of secondary structure in Cyc2$_{PV-1}$, the Cyc2 sequence from *M. ferrooxydans* PV-1 (D. W. A. Buchan, D. T. Jones, Nuc Acids Res, 2019, https://doi.org/10.1093/nar/gkz297).
